# The Modular Nature of Dendritic Cell Responses to Commensal and Pathogenic Fungi

**DOI:** 10.1371/journal.pone.0042430

**Published:** 2012-08-03

**Authors:** Lisa Rizzetto, Sonja I. Buschow, Luca Beltrame, Carl G. Figdor, Stephan Schierer, Gerold Schuler, Duccio Cavalieri

**Affiliations:** 1 Department of Preclinical and Clinical Pharmacology, University of Florence, Florence, Italy; 2 Translational Genomics Unit, Oncology Department, Mario Negri Research Institute, Milan, Italy; 3 Department of Tumor Immunology, Nijmegen Centre for Molecular Life Sciences, Radboud University Nijmegen Medical Centre, Nijmegen, The Netherlands; 4 Department of Dermatology, University Hospital Erlangen, Erlangen, Germany; 5 Research and Innovation Center, Fondazione Edmund Mach, via Edmund Mach 1, San Michele all’Adige (TN), Italy; McGill University, Canada

## Abstract

The type of adaptive immune response following host-fungi interaction is largely determined at the level of the antigen-presenting cells, and in particular by dendritic cells (DCs). The extent to which transcriptional regulatory events determine the decision making process in DCs is still an open question. By applying the highly structured DC-ATLAS pathways to analyze DC responses, we classified the various stimuli by revealing the modular nature of the different transcriptional programs governing the recognition of either pathogenic or commensal fungi. Through comparison of the network parts affected by DC stimulation with fungal cells and purified single agonists, we could determine the contribution of each receptor during the recognition process. We observed that initial recognition of a fungus creates a temporal window during which the simultaneous recruitment of cell surface receptors can intensify, complement and sustain the DC activation process. The breakdown of the response to whole live cells, through the purified components, showed how the response to invading fungi uses a set of specific modules. We find that at the start of fungal recognition, DCs rapidly initiate the activation process. Ligand recognition is further enhanced by over-expression of the receptor genes, with a significant correspondence between gene expression and protein levels and function. Then a marked decrease in the receptor levels follows, suggesting that at this moment the DC commits to a specific fate. Overall our pathway based studies show that the temporal window of the fungal recognition process depends on the availability of ligands and is different for pathogens and commensals. Modular analysis of receptor and signalling-adaptor expression changes, in the early phase of pathogen recognition, is a valuable tool for rapid and efficient dissection of the pathogen derived components that determine the phenotype of the DC and thereby the type of immune response initiated.

## Introduction

Only in the past decade has it become clear that the innate immune system not only specifically recognizes various classes of microorganisms, but also initiates and modulates the subsequent adaptive responses carried out by T cells and B cells. The type of adaptive immune response that follows microbial invasion is largely determined at the level of the antigen-presenting cells, and in particular by dendritic cells (DCs) which because of their unique antigen processing and presenting capacities are considered the professional antigen-presenting cells of the immune system. DCs contain a large array of pattern-recognition receptors (PRRs), which recognize conserved microbial components called pathogen-associated molecular patterns (PAMPs) and upon binding start a specific transcriptional program that activates the DC, consequently directing the immune response suited to face the recognized pathogen. One group of PRRs is formed by the C-type lectins that include the DC-specific ICAM-3 grabbing non-integrin (DC-SIGN) [1], [2] and DC-associated C-type lectin-1 (Dectin-1; CLEC7A) [3], [4] that both shape immune responses against various pathogens [3], [5], [6], [7], [8], [9], [10], [11]. The intracellular signaling pathways induced by these C-type lectins modulate the responses of other PRRs such as TLRs, but also exert independent and peculiar functions [12].

Engagement of different PRRs by the host immune surveillance results in complex signaling and determines subsequent fungal antigen processing and antigen presentation, contributing to the disparate patterns of reactivity observed locally in response to fungi [13], [14]. Pathogens mask cell wall components to circumvent processing and presentation [15], [16], and use C-type lectin receptors to escape immune activation.

*Candida albicans* and *Saccharomyces cerevisiae* are ubiquitous fungal organisms that often colonize the skin and mucosal surfaces of normal individuals, without causing disease. However, when normal host defense mechanisms are impaired, these yeasts may become pathogens. Putative virulence factors of *C. albicans*, include the ability to switch between being a saprophytic yeast and a filamentous pathogenic form of the fungus. DCs sense both forms in a specific way, resulting in distinct, T helper (Th)-cell-dependent protective and non-protective immunities respectively [14]. Recent evidence suggests that the use of distinct PRRs contributes to the differences in responses to diverse forms of *C. albicans* as well as of *S. cerevisiae*, including differential use of Dectin-1 [7], [17], [18], DC-SIGN and Mannose Receptor (MR) [19].

Successful resolution of pathogenic fungal disease depends on proper coordination of multiple components of the host immune response. Combating fungal infection requires a sophisticated understanding of the molecular mechanisms involved in pathogen recognition and in the host immune response. Widespread use of microarrays has generated huge amounts of data on the DC response to pathogens and the interrogation of public microarray repositories, where much of this data is contained, is now a major challenge, as is identifying precisely the sequence of events.

Unlike traditional biological research which isolates and studies a small set of components involved in a biological processes, systems biology takes on the complexity through study of the different components in parallel by the power of functional genomics. Systems biology involves acquiring information about the different levels of regulation of a biological system and we have applied this here to unravel the complexity and dynamic nature of the host-fungus interaction. The immunological complexity is not simply the product of a series of discrete linear signaling pathways; rather it is composed of complex networks of thousands of separately regulated molecules. DCs are particularly well suited to infer the system response to stimuli because of their great alteration of gene expression upon activation [20]. The highly dynamic spatio-temporal integration of signals may coordinate the regulation of RNA levels resulting in specific cytokine expression changes. This reflects profound cellular plasticity at the protein and metabolite level, which finally determines the phenotype of the cell and outcome of the immune response [21].

Recently, a combined forward and reverse genomics approach has allowed the reconstruction of the transcriptional and regulatory network driving the immune response in DCs [20]. This study lead to the identification of single regulators which control genes involved in inflammatory and viral responses depending on the differential timing, level and combination of regulator activation.

In this study we present expert annotated novel pathways for signaling in response to the DC-SIGN and Dectin-1 in DCs, both important players for the recognition of fungi. Using the newly curated pathway set of DC-ATLAS [22] we illustrate the modular DC response to different fungi and the contribution of single cell wall components in the induction of an immune response tailored to each specific invading fungus.

## Results

### Curation of DC-SIGN and Dectin-1

**Figure 1:**
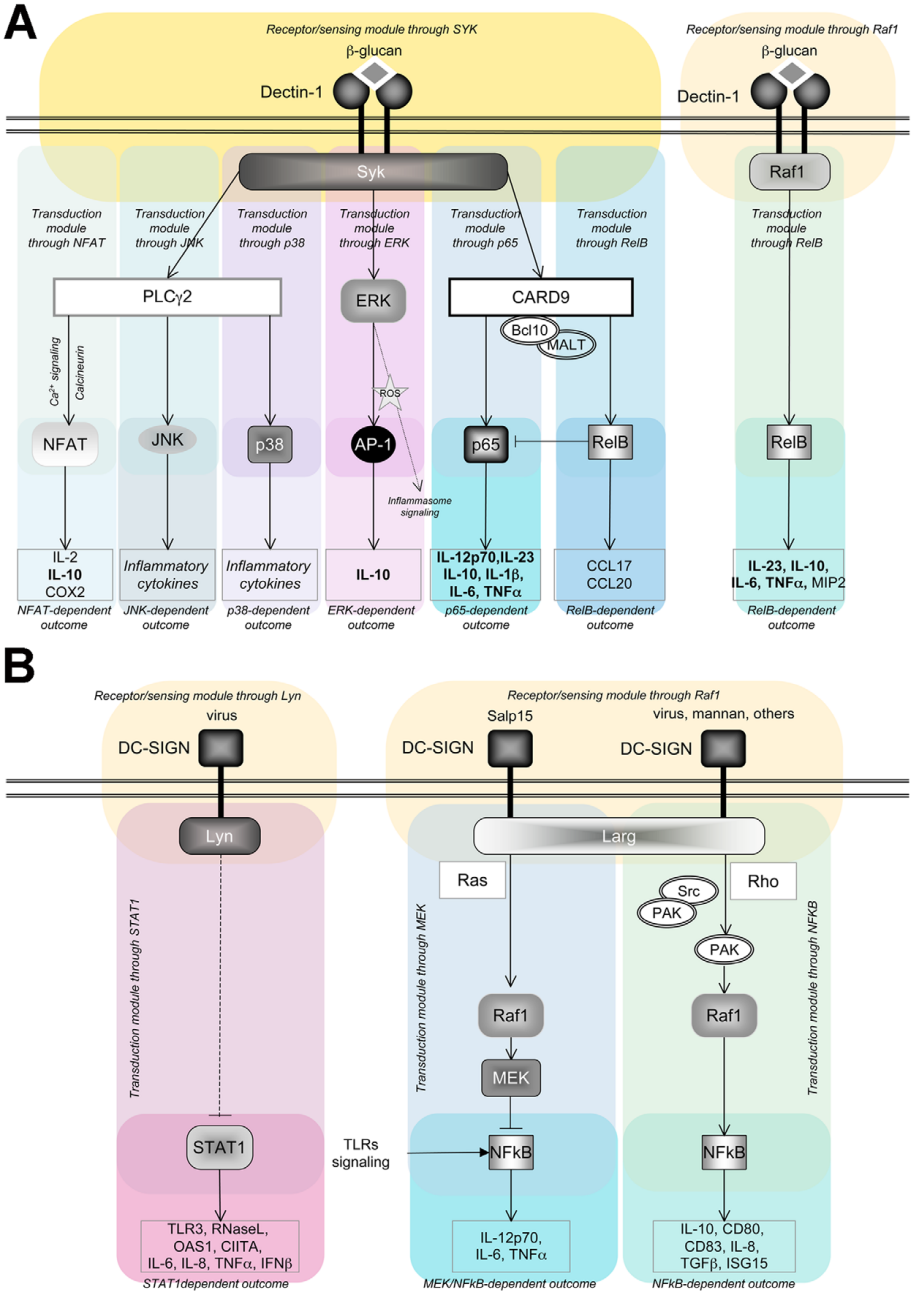
Flowchart of DC-SIGN and Dectin-1 signalling. Following DC-ATLAS pathway structure, receptor/sensing modules, transduction modules and the outcome modules were represented for (A) Dectin-1 signaling and (B) DC-SIGN signaling. For the outcome modules the cytokines measured within the manuscript are indicated (bold). These graphs present both the vertical and horizontal resolution of DC-TLAS pathways. For the complete BCML pathways, please see Figure S1 and S2. Note: the TLP1-mediated signalosome upon DC-SIGN engagement recently described has not been drawn to simplify view of the modules.

The curation process of the two pathways was started by collecting available literature to reconstruct the signaling cascade from the receptors to the genes transcribed in response to the various ligands so far described for DC-SIGN and Dectin-1. The pathway curation was performed using the Biological Connection Markup Language (BCML) [23] according to the guidelines developed by the DC-ATLAS initiative [22]. For DC-SIGN we defined (Figure S1) a modular structure composed of two receptor/sensing modules (“s” modules), based on two known adaptors for DC-SIGN - LARG and LYN - resulting in two transduction modules (“t”) and three outcome modules (“o”). In the same way, we defined for Dectin-1 (Figure S2) two receptor/sensing modules – driven by SYK and RAF1-, five transduction modules representing Dectin-1 activation of NFAT, ERK, p38, JNK and NFkB, and five outcome modules. Figure 1 is a flowchart of the signaling in the two pathways, depicting both the vertical and horizontal resolution of DC-ATLAS pathways (for detailed maps see Figure S1 and S2).

### Modular Pathway Analysis Allows Distinguishes Different DC Response to Fungi

**Figure 2:**
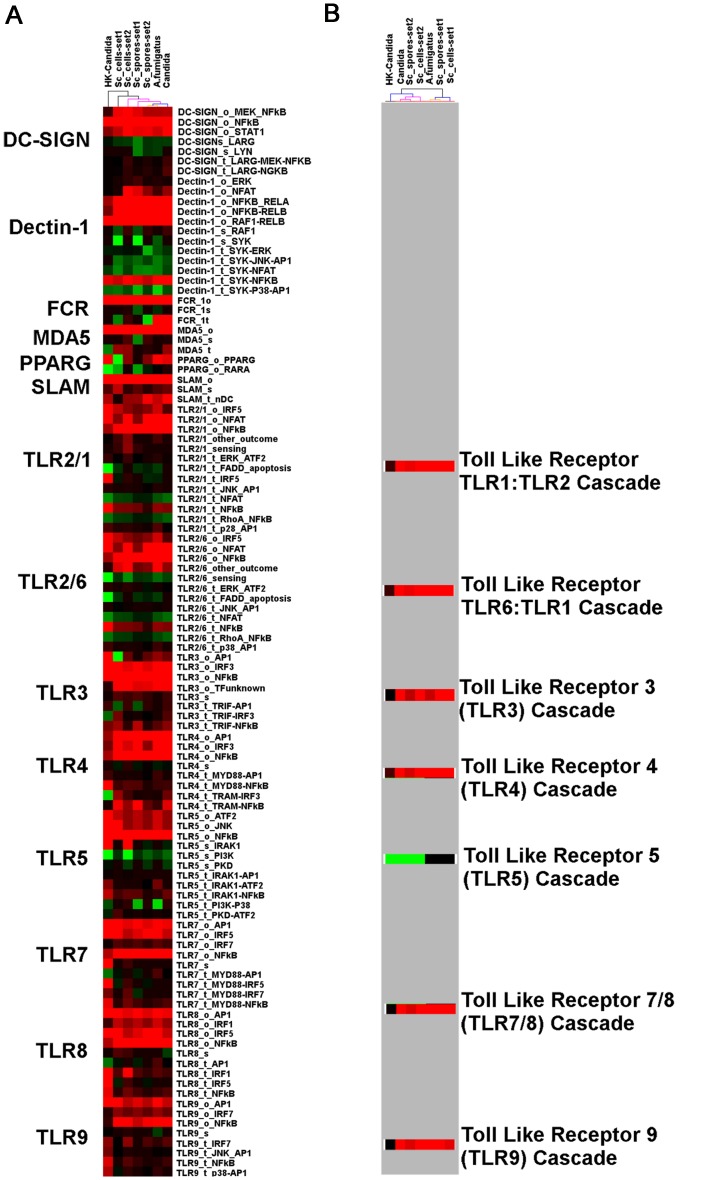
DC-driven signaling response upon fungal receptor engagement. Clustering of pathway Enrichment Factor (PEFs) obtained from the FET analysis on DC samples challenged with fungi. (A) Pathway signatures generated on DC-ATLAS. (B) Insert of the pathway signature generated using immune-related public pathway available from KEGG and Reactome. The detail highlights the available pathway involved in the initial recognition of an antigen, in particular the TLR signalings. The complete clustering is available as Figure S3. Colored spots indicate significant (p≤0.05) up- (red) or down- (green) regulation. The colors of the dendrogram indicate the percentages of the tree support (significance), from 50% (pink) to 100% (black).

In order to understand the response of DCs to fungi, we derived pathway signatures (see Material and Methods section), using the DC-ATLAS pathway set on five new or existing microarray data sets (Table 1; Table S1), including DCs stimulated with the supposedly harmless *S. cerevisiae* (Sc, both in the form of cells and of spores) and DCs stimulated with opportunistic pathogens, such as hyphae of *C. albicans*, both live or heat-killed (HK), and conidia of *A. fumigatus*. Resulting pathway signatures were clustered (Figure 2A). To improve the specificity of the analysis and to reduce the platform and donor variability effects, signatures were generated on the mean expression ratios per data set (average of replicates for each condition per data set). Clustering grouped the different fungi according to the pathway signatures of the transcriptional responses elicited in DCs. The disposition of samples in the cluster tree suggests that DCs respond in a specific manner to pathogenic or non-pathogenic fungi. In particular we confirmed that Sc spores [19], induced a DC response closer to that of *A. fumigatus* and *C. albicans* hyphae (Figure 2A). Interestingly, HK *Candida* stimulated samples were outside the cluster of live *C. albicans* stimulated sample. This likely reflects changes in exposure of cell wall components as a consequence of heat killing (Figure 2A). Thus, DC gene expression and derived pathway signatures by DC-ATLAS can be used as readout for fungal cell wall properties and to get insight into the type of fungal stimulus.

**Table 1:** Microarray experiments on DCs challenged with fungi and single agonists.

Dataset	Treatment	Short name reported in the work	Replicates for each treatment	Origin	Time point	Platform
E-MEXP-1745	1. Live *S. cerevisiae* yeastcells2. Live *S. cerevisiae* spores3. unstimulated cells	Sc cells – set 1, Sc spores – set 1	3 different donors	Cavalieri’s lab [40]	4 hours	Affymetrix
E-MTAB-135	1. Live *S. cerevisiae* yeastcells2. Live *S. cerevisiae* spores3. Live *C. albicans* hyphae4. unstimulated cells	Sc cells – set 2 Sc spores – set 2Candida	4 different donors	Cavalieri’s lab [19]	4 hours	Illumina
E-MTAB-751	1. Heat killed *C. albicans*hyphae2. unstimulated cells	HK Candida	4 different donors	Schuler’s lab – this work	3 hours	Illumina
E-GEOD-6965	*A. fumigatus* conidia	A. fumigatus	2 different donors	Loeffler’s lab [33]	6 hours	Affimetrix
E-MTAB-448	1. Resiquimod2. LPS3. unstimulated cells	R848 LPS	3 different donors	Cavalieri’s lab [22]	3 hours	Illumina
E-MTAB-750	1. Live *S. cerevisiae* yeastcells2. Live *S. cerevisiae* spores3. Curdlan4. Zymosan5. Mannan6. unstimulated cells	Sc cells Sc spores Curdlan Zymosan Mannan	4 different donors	Cavalieri’s lab – this work	4 hours	Illumina
E-MTAB-750	1. Curdlan2. unstimulated cells	Curdlan 0′Curdlan 5′Curdlan 15′Curdlan 30′Curdlan 60′Curdlan 120′	3 different donors	Cavalieri’s lab – this work	0, 5, 15, 30. 60. 120 minutes	Illumina

The microarray datasets used in the manuscript are directly accessible from http://dc-atlas.net/datasets/and Array Express (www.ebi.ac.uk/arrayexpress/experiments).

In contrast, when performing pathway analysis on publicly available pathways, the differences in the DC response to the diverse fungi were not completely discernible (Figure 2B). Even though the information provided by the immunologically-related-public pathways is complementary to that stored in DC-ATLAS, using publicly available PRRs pathways, we could not infer any indication about the receptor from which the induced signaling originated (Figure 2B; for the complete cluster with the public available pathways see Figure S3) indicating that the modular structure of the DC-ATLAS pathways greatly improved the resolution of the analysis.

The activation states of the various DC-ATLAS pathway modules showed a number of effects present across all data sets. These effects likely describe a common response to fungi that is irrespective of whether the fungus is pathogenic or not. Overall, datasets showed significant enrichment in genes of pathways specifically involved in fungal recognition such as DC-SIGN, Dectin-1 and TLR 2/6 signaling (Figure 2A).

Upon stimulation with non-pathogenic yeast the receptor/sensing modules of Dectin-1 and TLR2/6 pathways were mostly down-regulated. Also, part of the transduction modules of these pathways was either down-regulated or only weakly activated. DC-SIGN receptor/sensing module was down-regulated for all the data sets tested. The regulation of TLR7/8 suggests that signaling from antigens, released upon fungal processing in the endosome, converges and enhances the signal transduction cascade.

### Dissection of Fungal Recognition into Pathways Downstream of Specific Cell Wall Components

**Figure 3:**
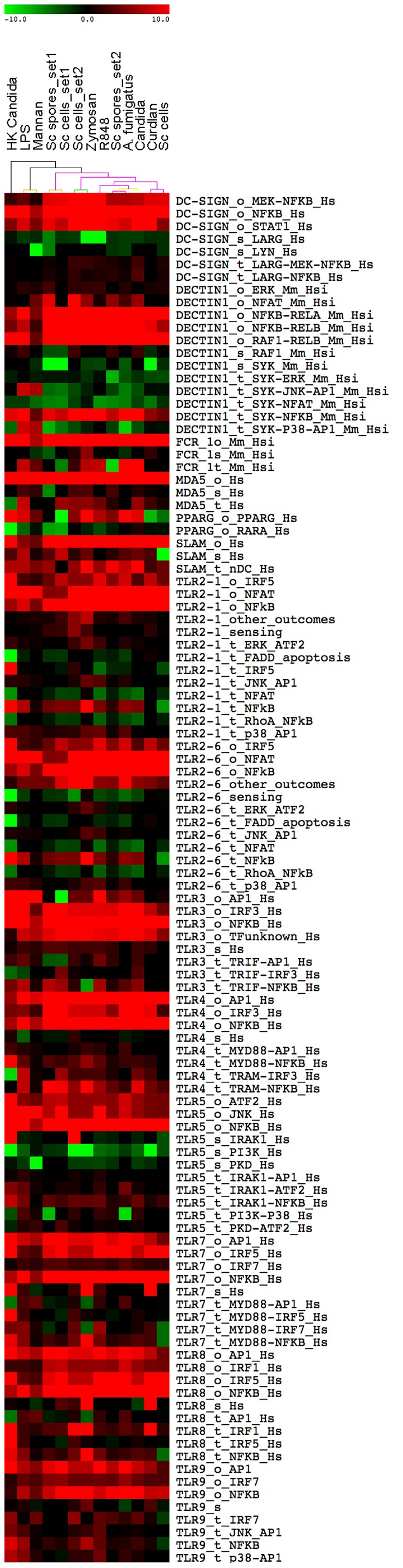
DC-driven signaling response upon fungi, C-lectin and TLR agonists. Transcriptional analysis was performed on DCs after 4 hours of stimulation with Curdlan, Zymosan, Mannan, Sc cells and spores or without any stimuli. Clustering of Pathway Enrichment Factors (PEFs) were obtained from the FET analysis of challenged DC samples and compared with those obtained on DC stimulated with whole fungi or the publicly available dataset of LPS or R848 stimulated DCs. Colored spots indicate significant (p≤0.05) up- (red) or down- (green) regulation. The colors of the dendrogram indicate the percentages of the tree support (significance), from 50% (pink) to 100% (black).

Fungal recognition is not the result of one microbial component interacting with a single receptor, but of a complex network of interacting receptors and ligands. The availability of ligands may be crucial in determining the DC response to different fungi. We hypothesized that DC-ATLAS was now able to distinguish pathogenic from non-pathogenic fungi because of its modular structure that can also detect more subtle differences in PRR receptor recognition. To investigate this in more detail we compared the DC responses to whole microorganism and fungal cell wall components by performing additional microarray experiments, stimulating DCs with more defined components of the fungal cell wall. In particular, we focused on the components of the *S. cerevisiae* (Sc) cell wall, which includes β-glucans and mannan. We performed a transcriptional analysis on human monocyte-derived DCs from four healthy donors stimulated with (a) Zymosan A, cell wall extract from Sc exposing mostly β-glucans and mannan, (b) with Curdlan, a β-glucan preparation, (c) with Mannan derived from Sc and (d) with the whole fungus, represented by Sc cells or spores themselves (Table 1). In addition, to compare the results with single TLR agonists and to test the specificity of our observations, we generated pathway signatures from publicly available data sets of DCs stimulated by the TLR4 agonist lipopolysaccharide (LPS) and the TLR7/8 agonist Resiquimod (R848). We then clustered the obtained Pathway Enrichment Factors (PEFs) with the data produced in-house (Figure 3).

We observed a common core of inflammatory response, but DCs stimulated with Mannan (thus without β-glucan) showed a different pathway profile than the rest of the fungi, and grouped together with LPS and Sc cells and remained distant from the live and most pathogenic fungi (Figure 3). The close association between LPS and Mannan is in agreement with previous studies indicating that the Mannan-LBP complex is recognized by the CD14/TLR4 signaling leading to the production of proinflammatory cytokines in a manner similar to that induced by LPS [24]. In contrast, the R848 sample clustered together with the whole fungal samples suggesting the DCs may respond to released RNA from phagocytosed fungi. That the endosomal pathway of TLR7/8 receptor was differentially regulated only upon administration of more complex stimuli and not by a single cell wall component demonstrates the importance of studying the response to whole fungal cells.

Curdlan signaling resulted in repression of the genes for the receptor module of Dectin-1 and Zymosan down-regulated DC-SIGN and Dectin-1 sensing modules while activating TLR2 and TLR4 pathways. A number of other receptor pathways were regulated by the stimuli. Several of the TLR pathways overlap in their signaling elements and therefore changes in these pathways do not necessarily mean these receptors are specifically involved (Figure 3). Interestingly, the most pathogenic fungi clustered most closely to the Dectin-1 specific ligand, Curdlan, and much less with LPS/Mannan, suggesting a shift in the balance between Dectin-1 and TLR4 signaling – or even a lack in their cross-talk- which may be correlated with the DCs response to fungi with a different level of pathogenicity.

We could not obtain the same results using publicly available pathways, in a similar manner to our previous pathway signatures on whole fungi (Figure S4). When using these pathways the differences in the specific signaling triggered by the diverse stimuli were not discernible.

**Figure 4:**
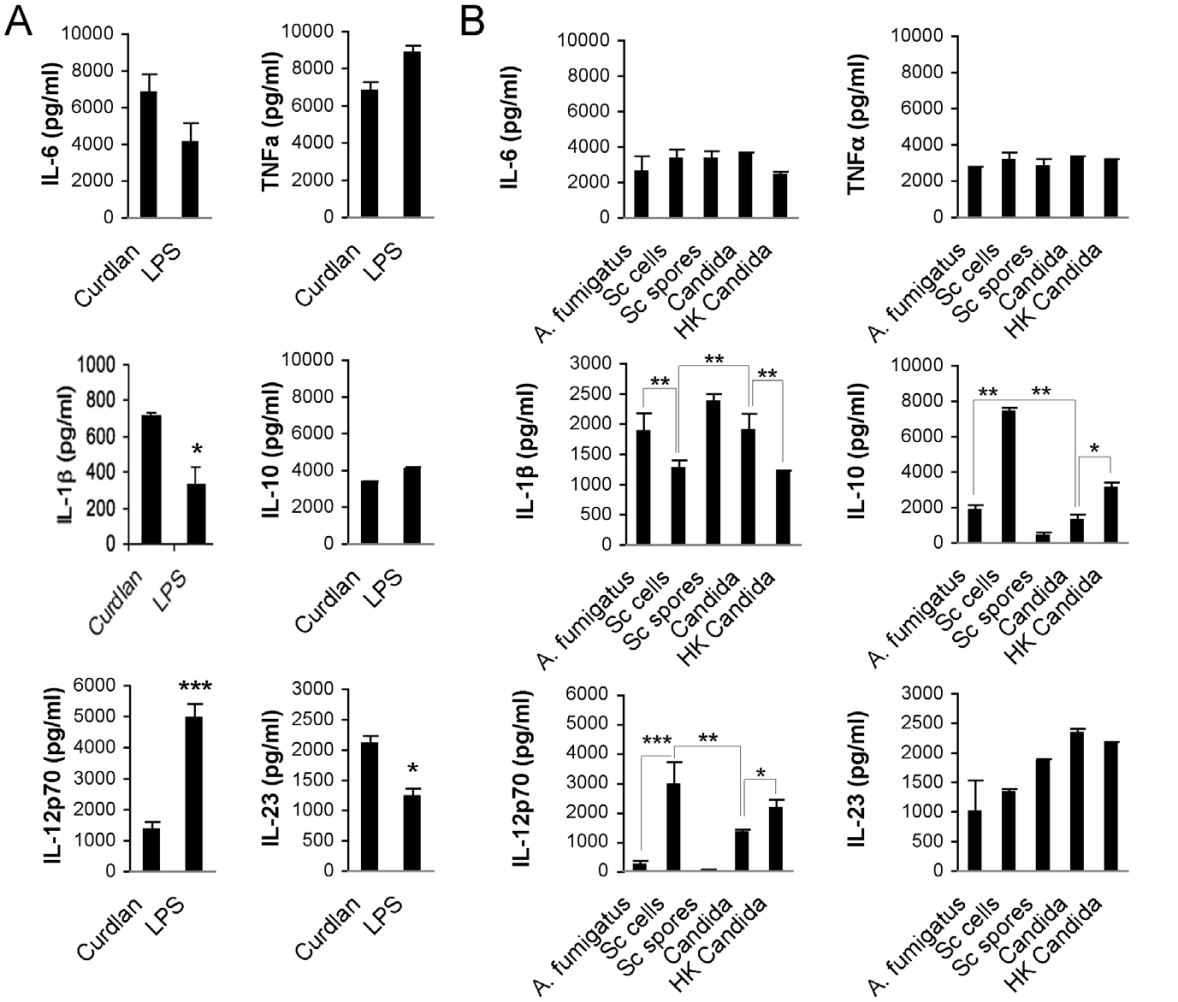
Differences highlighted in pathway analysis are revealed in cytokine induction. Cytokines profiles confirm the differences in immune recognition. (A) Cytokines production upon CLR and TLR agonist engagement. DCs were stimulated with Curdlan, LPS or without any stimuli for 18 hours. The level of IL-1β, TNFα, IL-12p70 and IL-6 production was measured using the Milliplex® technology. (B) Cytokine level detection on DC supernatants upon stimulation with the live *Candida* or Sc cells for 18 hours. Detection was performed as mentioned above. **p<0.01, ***p<0.001.

Encouraged by our results, we hypothesized that the pathway signature may be translated to a cytokine profile reflecting the relative importance of the receptors, as revealed by our pathway analysis. Although many cytokines are in principle shared by several pathways we reasoned the balance between the cytokines may be quite specific for each stimulus and would thus allow us to discern the contribution of certain receptors. To test this we first compared the cytokines profiles triggered by the receptors that seemed best able to distinguish the DC response, TLR4 and Dectin-1 by the single components LPS and Curdlan respectively (Figure 4A and Figure S5). Curdlan mostly triggered IL-1β whereas LPS most specifically induced IL-12p70. Minor differences were also visible for the other cytokines but less marked. Thus IL-1β and IL-12p70 may now be used as an indication of the contribution of Dectin-1 and TLR4 in the recognition of pathogenic *vs*. non-pathogenic fungi. Indeed, live *Candida* produced more IL-1β whereas the harmless Sc cells produced relatively more IL-12p70 and IL-10 than the pathogenic fungi. Similarly to Sc cells, HK *Candida* showed less IL-1β and higher IL-12p70 suggesting again a switch in receptor balance or the availability of ligands over time.

### Timing and Involvement of Dectin-1 in Fungi Recognition

Our results have thus far demonstrated differential clustering of pathway signatures for the stimuli tested, suggesting differences in the interaction of pathogenic versus non-pathogenic fungi with Dectin-1 and TLRs. As a follow-up to our initial analysis, we investigated whether pathway signatures could provide a deeper insight into the differences at the receptor level. In particular, we took a closer look at the Dectin-1 receptor, which is engaged by *S. cerevisiae* and the fungal pathogens we took into account. The Dectin-1 pathway is composed of a receptor/sensing module (represented with an “s”) various transduction modules (represented with an “t”) that all result in a specific outcome (represented with a “o”). The Dectin-1 receptor/sensing module (represented with an “s”) exhibited different regulation between live *C. albicans* and the rest of the samples: this module was not significantly changed for *C. albicans*, while for the other samples it showed a clear down-regulation (Figure 2 and 3) suggesting a feedback down-regulation of the receptor sensing module. Possibly this is due to a difference in signal strength. Indeed, the dataset we used derived from DCs stimulated with live *C. albicans* hyphae and the lack of effect on the Dectin-1 receptor/sensing module may relate to the notion that less Dectin-1 ligand is exposed on the filaments of *C. albicans* as compared to the yeast form [7].

The outcome modules of Dectin-1 are significantly up-regulated, indicating that, at this time point, a large part of the transduction signal has already propagated to the final part of the pathway. In addition to the Dectin-1 pathways, our findings indicate that genes present in the outcome modules of DC-SIGN in general exert an important role in the recognition of entire fungal cells, consistent with what was previously reported at the protein level [25], [26], [27].

**Figure 5:**
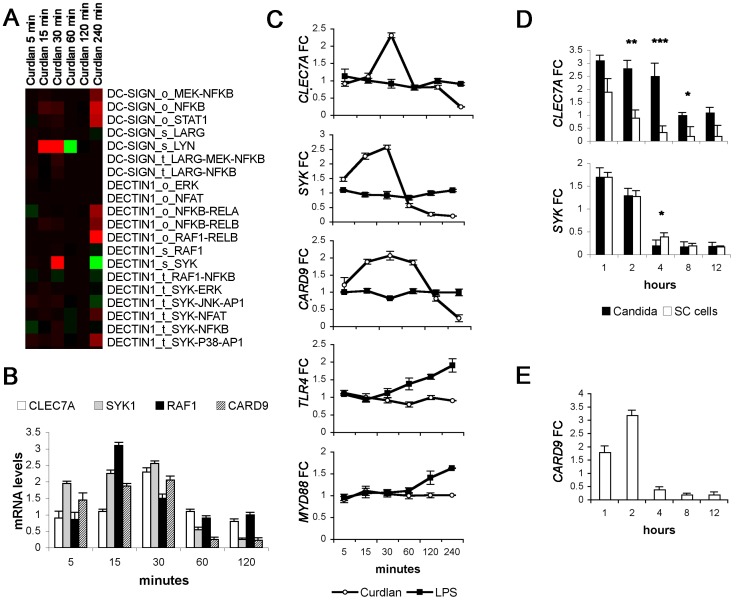
Dectin-1 modular structures in response to ligand concentration determine differences in fungi recognition. (A) Clustering selection of Pathway Enrichment Factor (PEFs) obtained from the FET analysis on DC samples stimulated with Curdlan in time. Colored spots indicate significant (p, 0.05) up- (red) or down- (green) regulation. The colors of the dendrogram indicate the percentages of the tree support (significance), from 50% (pink) to 100% (black). (B) *CLEC7A, SYK, RAF1* and *CARD9* gene expression on DCs upon Curdlan stimulation. Gene expression was assessed by RT- PCR on DCs stimulated with Curdlan for 5, 15, 30, 60 and 120 minutes. (mean ± SD, N = 3). (C) DC modulation of gene expression of CLR or TLR signalling. The specificity of signals to different PRR agonists was assessed by real-time PCR on DCs stimulated with Curdlan or LPS for 5, 15, 30, 60, 120 and 240 minutes (mean ± SD, N = 3). (D) The differences at the sensing level, between *C. albicans* hyphae and Sc cells stimulation were addressed by RT-PCR. *CLEC7A* and *SYK* gene expression on DCs upon challenge with *C. albicans* hyphae and *S. cerevisiae* cells was assessed on DCs stimulated for 1, 2, 4, 8 and 12 hours (mean ±SD, N = 3): **p<0.01, ***p<0.001. (E) *CARD9* gene expression on DCs upon challenge with Sc cells. Gene expression was assessed in RT-PCR on DCs stimulated over time (mean ± SD, N = 3); FC, fold change was calculated by comparing the stimulated condition at various time points with the unstimulated control after normalization to the expression of the houskeeping gene GAPDH.

Thus, at 4 hours after stimulation, most signals had already produced changes up to the outcome modules. To test whether the DC-ATLAS modular structure would also allow us to follow signal propagation from receptor sensing to outcome we took a specific look at the Dectin-1 pathway modules over time using Curdlan, the Dectin-1 ligand. To make the flow through the modules visible we studied the response to Curdlan also for shorter time-points (Figure 5A, for the complete cluster please see Figure S6). Consistent with the modular structure of DC-ATLAS, Curdlan at 30 minutes first triggered changes in the sensing modules of the Dectin-1 pathway whereas effects in the transduction and outcome modules were most apparent at 4 hours in line with the need for transcriptional regulation of cytokines and chemokines coding genes. The signal flow can thus be readily followed by mapping the affected genes present in the pathway signatures in the respective pathway modules (Figure S7, S8, S9, S10, S11, S12, S13).

Intriguingly, Curdlan also triggered the Lyn-dependent module of the DC-SIGN pathway suggesting this module may be of importance for Dectin-1 signaling as well.

Consistent with our data on whole fungi we noticed that after an initial up-regulation of the receptor sensing and transduction modules of Dectin-1 these modules were down-regulated at later times suggesting negative feedback regulation after cell commitment.

To look further into this feedback process, we investigated the expression of several important players in Dectin-1-mediated recognition and signaling with real time PCR (RT-PCR). After stimulation with Curdlan, the mRNA levels of the gene coding for Dectin-1 (*CLEC7A*) and of its adaptors, the spleen tyrosine kinase (*SYK*), *CARD9* and *RAF1* were analyzed (Figure 5B). RT-PCR confirmed the pathway signalling flow and showed that after an initial up-regulation directly after signal initiation by curdlan the genes were down regulated. This type of transcript modulation however was specific for the Dectin-1 sensing module and not seen for TLR4 and its downstream signalling component MyD88 (Figure 5C). LPS up-regulated *TLR4* and *MYD88* genes after 60 minutes of stimulation and their expressions were sustained over time. In general, the level of signal transduction components seemed to vary over time. This also confirms that the results obtained from the pathway analysis are receptor-specific and they do not derive from cross-talk of different, unrelated signals.

Our 4 h-microarray results also showed a down-regulation of the sensing and transduction modules after triggering the CLR pathways with whole fungi or single compound (Figures 2 and 3). To confirm this and to test whether differences may exist between *Candida* and the other fungi we performed a RT-PCR at 4 h and at earlier (1 and 2 h) and later time points (8 and 12 h) using whole live *Candida* hyphae or Sc cells as stimuli (Figure 5D). Indeed both the transcripts for Dectin-1 and its downstream adaptor Syk were, after an initial up-regulation, down-regulated: consistent with the microarray data this down-regulation was considerably slower for *Candida* and not yet apparent at 4-hours. RT-PCR also showed the down-regulation of the gene encoding the Dectin-1 adaptor CARD9 (*CARD9*) at later time points upon Sc cells-stimulation (Figure 5E).

Altogether these results indicate that soon after the initial burst in expression in response to fungal exposure, DCs start to down-modulate most of the receptor sensing modules responsible for fungal recognition and this process may be sped up by the number of receptor-ligand interactions, possibly determining the difference in timing of receptor regulation between the pathogenic *Candida* and the non-pathogenic Sc cells.

**Figure 6:**
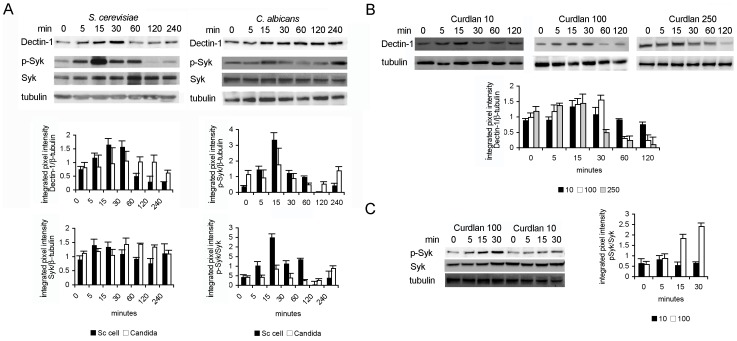
Immunoblot analyses of Dectin-1 and Syk upon fungal recognition. DCs were stimulated with live *S. cerevisiae* (Sc) cells, *Candida* (A) or different concentration of Curdlan (µg/ml, B, C) for the time indicated. Cell lysates were isolated and immunoblotted with the specific primary antibodies and reprobed with anti-β-tubulin antibody to control for the equal loading of cell lysates. Representative blots are shown. Quantitative analysis of protein expression is summarized in bar graphs, presented as mean integrated intensity of specific bands normalized to β-tubulin ± SD of 3–5 independent experiments.

To investigate this in more detail we looked at the regulation of Dectin-1 and down-stream signaling components at the protein level. Indeed we could confirm a down-regulation of Dectin-1 in response to Sc cells. Consistent with the RNA data, Dectin-1 was also decreased upon *Candida* recognition but at later time points (Figure 6A). A decrease was also seen when DCs were stimulated with Curdlan (Figure 6B) but the timing depended on the Curdlan concentration showing that the low availability of the Dectin-1 ligand on the *Candida* surface may not be sufficient to rapidly down-regulate the Dectin-1 sensing module. Consistent with this notion, Syk that acts directly downstream of Dectin-1, is quickly and highly phosphorylated upon stimulation with Sc cells and thereafter rapidly shut-off (Figure 6A). After *Candida* stimulation however this fast phosphorylation was less pronounced and progressed in a wave-like fashion consistent with a remaining presence of receptor signaling. Also for Syk phosphorylation the intensity was clearly concentration dependent (Figure 6C). Our RNA and protein data indicate that the slow release of β-glucans from *Candida* could pose kinetic differences in receptor regulation and activation that may contribute to the differential response of DC to *S. cerevisiae* and *Candida*.

### From Whole Genome to Single Genes

**Figure 7:**
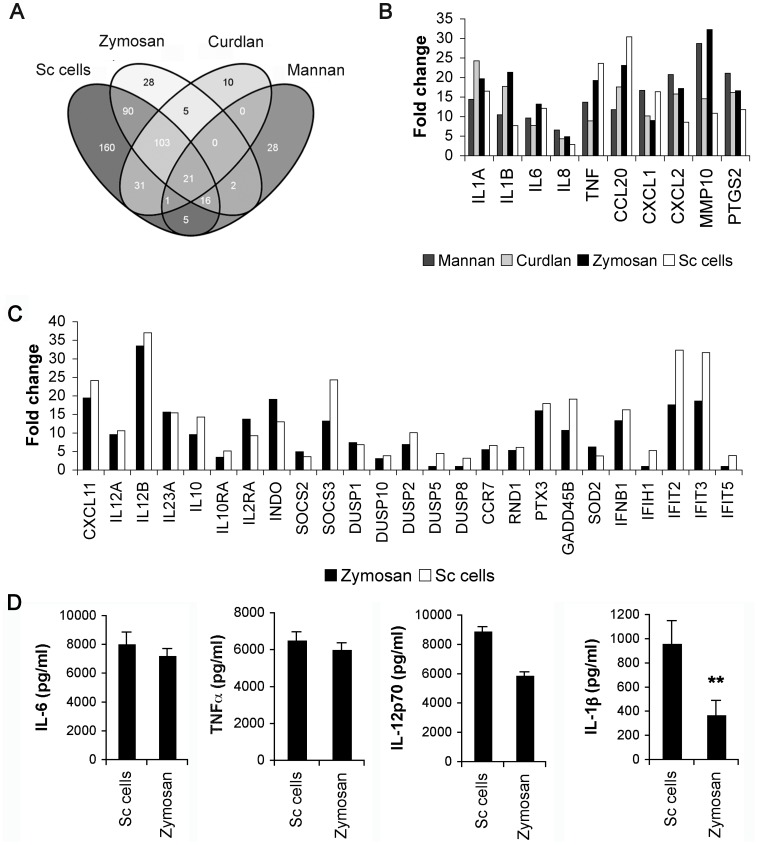
Immune response (IR) genes activated by different cell wall components and *Saccharomyces cerevisiae* in DCs. Transcriptional analysis was performed on DCs after 4 hours of stimulation with Curdlan, Zymosan, Mannan and *S. cerevisiae* yeast or without any stimuli. (A) Venn diagram of upregulated DEGs. (B) IR differentially regulated genes (p≤0.05) commonly expressed among the four stimulation conditions. (C) IR differentially regulated genes (p≤0.05) commonly expressed among Zymosan and *S. cerevisiae* yeast stimulation condition and not affected upon single cell wall components stimulation. (D) Cytokine detection assays were performed for IL-1β, TNFα, IL-6 and IL-12p70 measurement on supernatants of DCs stimulated for 18 hours with Sc cells or zymosan; **p<0.01.

Our analysis so far confirms that pathway signatures are a useful tool to detect general differences and commonalities between different datasets. This approach however is complementary to and should not abrogate the classical differential gene analysis, which provides a more detailed description of the precise nature of the immune response activated by the different fungal cell wall PAMPs at the single gene level. Unfortunately here we are restricted in our possibilities by the fact that only datasets obtained under the same conditions and using the same microarray platform can reliably be compared; a problem that can be overcome by using pathway signatures [28]. Nonetheless we could reliably compare the single differentially expressed genes (DEGs) on DCs stimulated with the different cell wall components or the whole fungus, each condition compared in triplicate (see Materials and Methods). We compared the single compounds - Curdlan and Mannan - to the more complex Zymosan and live Sc cells. We found, consistently with the pathway signatures, DCs stimulated with Curdlan or Mannan shared few responsive elements (22; Table S2). Zymosan and Sc cells shared differentially expressed genes with both Curdlan (129 genes; Table S2) and Mannan (39; Table S2). The effect of Zymosan and Sc cells was most complex and induced the differential expression of the largest number of genes, consistent with the notion that these contain ligands for multiple PRR (Figure 7A).

Despite a general inflammatory response (Figure 7B), we can deduce from the specifically changed genes that Zymosan and whole yeast, but not the single compounds (Mannan and Curdlan), were able to activate the expression of genes involved in Th1 response such as *IL12A* and *IL12B* genes (Figure 7C). However, despite the strong activation of Th1 polarizing factors, Zymosan-stimulated- and yeast-stimulated DCs also activated several genes that mediate immune-suppression (*IL10*, *IL-10R*, *SOCS2*, *INDO* and *IL2R*) (Figure 7C).

Live yeast cells up-regulated several genes associated with cell damage and repair that were either not or were less affected by Zymosan or single components (Figure 7C). Interestingly, Sc yeast, in contrast to the other compounds, was a stronger inducer of type I interferon and type I interferon associated genes (Figure 7C) suggesting activation of a more general state of alarm only when specific - or a combination of - signals are present.

In addition, whole yeast, unlike the single compounds, enhanced the secretion of IL-1β, confirmed at the protein level by ELISA (Figure 7D). Induction of biologically active IL-1β requires NF-*κ*B-mediated transcription of pro-IL-1β followed by caspase-1 cleavage of the inactive cytokine [29]. Caspase 1 in turn needs to be activated by the inflammasome [30]. Since we observed the overexpression of the purinergic ion channel receptor P2XR7 gene with transcriptional analysis (Figure 7C) uniquely after Sc stimulation, it is very well possible that this receptor is involved in the activation of IL-1β after stimulation of DC with yeast.

## Discussion

The immune response is a complex system with many possible inputs, interactions and outcomes. Structured analysis of the dataflow in these cells holds the promise of both better understanding this system and predicting the response of these cells to a given stimulus. Host/fungal interactions have so far been studied only for pathogenic fungi such as *C. albicans*, but it is not known what keeps commensal fungi far from being recognized as pathogenic. To elucidate the mechanism by which the host immune system discriminates friends from foes, we used the specifically tailored data model, the BCML [23] and the DC-ATLAS type of community curation [22] to annotate the modules that make the transcriptional network for the C-type lectins DC-SIGN and Dectin-1. DC-ATLAS pathways were then used to generate pathway signatures and to compare different microarray experiments in order to obtain a detailed outlook of the genomic response to different fungi or fungal forms over time. Our results show how the accurate annotation of existing knowledge on signaling by Dectin-1 and DC-SIGN can lead to the understanding of the DC response to similar microorganisms with a very different immunological outcome. We were not able to obtain the same results using traditional pathways in public databases. One reason is that core modules of the immune network crucial in this process, such as DC-SIGN and Dectin-1, simply are not available. Secondly, the other pathways (the public counterparts of the ones stored in DC-ATLAS) are not specific for a cell type, and as such contain connections that are absent in DCs, or miss components that actually play a role in the transduction mechanism. Finally, we were able to dissect the flow of information thanks to the structure of the DC-ATLAS, which describes pathways making the immune network as assemblies of three modules: sensing, signal transduction and outcome. This modular nature allows us to further distinguish responses into receptor-specific signaling pathways and with respect to the timing of the signal flow. Thus, DC-ATLAS pathways allowed us to differentiate the temporal and modular events characterizing the signaling cascades from initial fungal recognition to DC maturation. The results of the analysis are thus much improved with respect to the results one can obtain from existing pathway databases, which cannot significantly sort out changes in specific processes because they are too large and redundant. In general, our results demonstrate significant added value when re-analysing already available datasets with this newly designed tool.

Our pathway analyses indicated that the different immune response to Sc yeast cells and *Candida* hyphae stems from an important difference in the impact on transcription of C-type lectin pathway components. This evidence supports the observation that the activation of the Dectin-1 pathway is probably strongly delayed after stimulation with *Candida* hyphae possibly due to a slower ligand release and resistance to degradation in the endosome. Indeed *Candida* hyphae do not readily expose β-glucans on their surface but these may be released slowly upon phagocytic processing leading to low but sustained Dectin-1 signaling. On the other hand, *S. cerevisiae* cells will signal strongly through Dectin-1 from the moment of initial recognition. Not only the amount of ligand available but also the timing of its release and the location (e.g. plasma membrane or phagosome) may determine the outcome. Our data suggest that the regulation of receptor signaling both in place and over time may balance tolerogenic and inflammatory responses.

Efficient recognition of the stimulus results in an active down-tuning of the receptor as exemplified by the shut-down of Dectin-1 (*CLEC7A* gene) expression. This is a new finding, whose statistical assessment is totally dependent on use of the DC-ATLAS logic. The down-regulation of the C-lectin receptor modules as early as 4 hours after stimulation suggests that, at that time point, the cell is already dedicated to priming its response towards fungi. This indicates that the receptor mRNA levels are important for cell homeostasis and that the protein levels are tightly and rapidly controlled by efficient turnover mechanisms. The significant down-regulation, we reported, is in agreement with previous observations that the expression of C-type lectins is highly regulated by maturation stimuli, leading to their down-regulation as DCs mature [31].

Through the use of DC-ATLAS we were able to discern the differential involvement of receptors that relate to differences in the disposition of cell wall components (i.e, *C. albicans* hyphae vs Sc yeast cell form), as well as the possibility of distinguishing pathogenic from non-pathogenic fungi. Also, we were able to extract the contribution of single cell components during the response to a whole fungus.

We demonstrated that the integration of multiple stimuli over a defined temporal window is required to obtain an effective response.

In line with the known complex organisation of the fungal cell wall, our analysis reveals how fungal recognition is not simply the product of a series of linear signaling pathways but it is comprised of a complex set of integrated responses arising from a dynamic network of thousands of molecules that are subject to regulatory mechanisms themselves.

We highlight the ability of phagocytosed *S. cerevisiae* to induce a broader and more intense innate immune response than when different glycans are engaged to their corresponding PRRs alone. Overall, these core responses suggest that it is really the integration of signals, from both TLR and C-type lectins that determines the response to yeast cells rather than a response dominated by one or only few receptors.

Altogether our results show that the signal travels rapidly through the network and affected DCs are committed promptly after signal initiation when DCs rapidly down-regulate their receptor sensing modules and elicit signals stimulating downstream immune effectors depending on ligand concentration and/or availability. Differential activation of DCs by the various fungal stimuli results in different cytokine expression profiles that are related to their pathway profiles. Thus, depending on the type and state of the fungus, DCs respond with different cytokine signals to instruct an adaptive T cell response tailored to that specific fungal threat.

This work is based on *in vitro* experiments. *In vivo* the situation is indeed more complex, with the interplay and cross-talk among different immune cells and stromal cells that together determine the final response to the pathogen. Nonetheless we demonstrate that differences between pathogenic or harmless fungal strains exist when it comes to detection by DCs, important players in the initiation and control of the immune response. These findings should be expanded to study of the response of other immune or non-immune cells that also come into contact with these pathogens and that possess similar or other types of pattern recognition receptors.

This work establishes a broadly applicable approach, instrumental in revealing transcriptional and regulatory networks of immune cells upon pathogen recognition, leading to a better understanding of fungal infection in general and in relation to host genetic susceptibility. A better understanding of the modular nature of DCs response to fungi will only come from comparative studies of the immune response to whole cells of pathogenic and non-pathogenic species and strains. Only then we will fully understand how the host immune system detects pathogens.

## Materials and Methods

### Ethics Statement

The experimental plan was approved by the local Ethical Committee of Azienda Ospedaliera Universitaria Careggi (AOUC, Firenze, Italy), and written informed consent was obtained from all donors (Rif. n. 87/10).

### DC Preparation and Stimulation

PBMCs were isolated from buffy coat blood samples from healthy donors from the Transfusion Unit of the Careggi Hospital (Firenze, Italy) by Ficoll-Hypaque density gradient centrifugation (Biochrom AG). Monocytes were isolated from low density PBMCs by magnetic enrichment with anti-CD14 beads (Miltenyi Biotec). Cells were cultured in the presence of GM-CSF (800 U/ml) and recombinant IL-4 (1000 U/ml) for 6 days to allow DC differentiation [32]. Depending on the experiments, DCs were co-cultured in the presence of the specific stimulus.

For fungal stimulation, we prepared hyphae of SC5314 *C. albicans* strain, spores or cells of SK1 *S. cerevisiae* strain and conidia of Af293 *A. fumigatus* strain as previously reported [19], [33]. *C. albicans* were inactivated by heat-killing at 65°C for 3 hour. The efficiency of the treatment was assessed by plating the sample on solid medium and counts the colony forming unit after 3 days of growth. The stimulation was carried out by adding the fungal preparation (fungus:DC ratio 4∶1) or the appropriate concentration of the single receptor agonists to the DCs.

### Cytokine Production

After stimulation with the appropriate stimuli, supernatants were collected at the time indicated and cytokines detected. Human Milliplex® assay for IL-1β, TNFα, IL-12p70, IL-23 and IL-6 production was performed according to the manufacturer’s instructions (Millipore) using the Luminex technology. Statistical significance was assessed with a paired t-test.

### Quantitative Real Time PCR

Total RNA was extracted with TRI Reagent (Sigma-Aldrich). Random hexamer and reverse transcriptase kit (SuperScript II, Invitrogen) were used for cDNA synthesis. Transcripts for *CLEC7A*, *CARD9*, *RAF1, SYK*, *TLR4, MYD88,* were quantified with Applied Biosystems predesigned TaqMan Gene Expression Assays and reagents according to the manufacturer’s instructions. Quantification of the PCR signals was performed by comparing the cycle threshold (Ct) value of the gene of interest with the Ct value of the reference gene *GAPDH*. Values are expressed as fold change (FC) of mRNA relative to that expressed in unstimulated cells. Statistical significance was assessed with a paired t-test.

### Transcriptional Analysis of DCs Exposed to Different Cells Wall Components as Well as Whole Fungi

2×10^6^ DCs were left unstimulated or were stimulated with cells of *S. cerevisiae*, spores, or heat-killed (HK) *Candida albicans* hyphae in a ratio of 4∶1 or with Mannan, Curdlan and Zymosan (100 µg/ml). After 3 (HK *Candida*) or 4 hours, cells were collected. For the time course experiment, 2×10^6^ DCs were stimulated with Curdlan (100 µg/ml) for 5, 15, 30, 60, 120 minutes, or without any stimuli. RNA preparation, labeling, hybridization on a HT12 WholeGenome BeadArray (Illumina), and scanning were performed according to the Illumina reference protocols. The analysis was performed on four different donors. Bead-summary data saved from Illumina BeadStudio was pre-processed in several steps. Firstly, the background signal was assessed and corrected using the intensity signal from the control probes present on the array, and then quantile normalization was performed. In addition to background correction, Illumina probe identifiers were converted to nucleotide universal IDentifiers (nuIDs) [34] specific for the nucleotide sequence of each probe. The computation was performed using the lumi package [35] written in the R programming language.

### Differential Expression and Annotation

Differential expression analysis was carried out on the in-house Illumina data using the Rank Product algorithm [36], taking into account the differences between donors. p-values estimating differential expression were corrected for multiple testing (FDR) and genes with a corrected p-value ≤0.05 were selected.

Differentially expressed gene lists from the various data sets were uploaded in the Database for Annotation, Visualization and Integrated Discovery (DAVID; http://david.abcc.ncifcrf.gov) to perform functional annotation. The up-regulated and down-regulated gene lists were annotated separately.

### Analysis of Data from External Repositories

An experiment on stimulation of DCs with germinating tubes of *A. fumigatus* was retrieved from GEO (E-GEOD-6965; Affymetrix format). Raw data (CEL files) were then background subtracted, normalized, and summarized using the Robust Multi-Chip Average method [37], re-annotating the probes with newer definitions [38]. After normalization, absolute-scale intensity values were transformed into paired stimulated DC-unstimulated DC log2 ratios and processed with the analysis pipeline.

### Pathway Signature Generation

Pathway signatures were generated according to the procedure by Beltrame et al. [28]. Briefly, the Fisher’s Exact Test was run on paired case-control ratios calculated from the normalized data, using the DC-ATLAS pathway set. p-values were then transformed into pathway signatures, either signed Binary Enrichment Factors (sBEFs) or Pathway Enrichment Factors (PEFs). The operation was carried out with a custom analysis pipeline. The results were then clustered using multiscale bootstrap resampling [39], with Euclidean distance and average linkage as parameters. The analyses were carried out on different dataset, three already public available and two newly produced.

The detailed dataset used are listed in Table 1 and are available at http://dc-atlas.net/datasets/and on Array Express (www.ebi.ac.uk/arrayexpress/experiments). In figures, the datasets presenting DCs stimulated with the same fungi were listed as set 1 and set 2.

#### Immunoblot Analysis

DCs were stimulated with *S. cerevisiae* or *C. albicans* for 0, 5, 15, 30, 60, 120, 240 minutes or with different concentration of Curdlan (10, 100 and 250 µg/ml) for 0, 5, 15, 30, 60 and 120 minutes. DCs were lysed on ice in RIPA buffer supplemented with a phosphatase inhibitor mixture (Sigma). Lysates were centrifuged at 15,000×*g* for 10 min and stored at −80°C for further analyses, and protein concentration was determined by the Bradford assay. Equivalent amounts of proteins were diluted in Laemmli sample buffer, heated to 90°C for 5 min, loaded on 4–10% SDS-polyacrylamide gels, and transferred onto PVDF membranes (Biorad). Membranes were blocked with 5% nonfat dry milk in Phospate-buffered saline containing 0.05% Tween 20 (PBS-T). Primary Abs were diluted in 1% milk solution, following the manufacter’s instruction. After incubations with primary Abs for 1 hour, the membranes were washed and incubated with the appropriate diluted 1∶1000 in PBS-T for horseradish peroxidase-conjugated secondary antibody 1 hour at room temperature then rinsed with PBS-T. Immunoblots were developed using an enhanced chemiluminescence detection system. When necessary, membranes were stripped by heating at 56°C in 62.5 mM Tris-HCl, pH 6.7, with 100 mM 2-mercaptoethanol and 2% SDS. Quantification of immune-reactive bands was obtained by densitometric analysis with the aid of ImageJ software. The primary antibodies used were: human Dectin-1/CLEC7A Ab (R&D Systems), phospho-Syk (pY525/526) mAb (clone EP575(2)Y, Epitomics), human Syk mAb (clone EP573Y, Epitomics), human β-tubulin mAb (clone 1G3, AbD Serotec), The appropriate secondary antibodies were purchased from R&D Systems.

## Supporting Information

Figure S1 **DC-SIGN map.** The curation process lead to the reconstruction of signaling pathways. The map was drawn in an SBGN format. See Beltrame et al., 2011 and Cavalieri et al., 2010 for further details.
(PDF)

Figure S2 **Dectin-1 map.** The curation process lead to the reconstruction of signaling pathways. The map was drawn in an SBGN format. See Beltrame et al., 2011 and Cavalieri et al., 2010 for further details.
(PDF)

Figure S3 **Clustering of Pathway Enrichment Factors (PEFs) obtained from the FET analysis on DC samples challenged with the different fungi.** Pathway signatures were generated using immune-related public pathway available from KEGG and Reactome. Colored spots indicate significant (p≤0.05) up- (red) or down- (green) regulation. The colors of the dendrogram indicate the percentages of the tree support (significance), from 50% (pink) to 100% (black).
(TIF)

Figure S4 **Clustering of Pathway Enrichment Factors (PEFs) obtained from the FET analysis on DC samples challenged with CLR-agonists.** Pathway signatures were generated using immune-related public pathway available from KEGG and Reactome. Colored spots indicate significant (p≤0.05) up- (red) or down- (green) regulation. The colors of the dendrogram indicate the percentages of the tree support (significance), from 50% (pink) to 100% (black).
(TIF)

Figure S5 **Changes in expression of cytokines at 4 h of stimulation.** Cytokine gene expressions on DCs upon 4 hour-stimulation with *A. fumigatus* conidia, Sc cells and spores, heat-killed (HK) or live *C. albicans* hyphae. Gene expression was assessed in RT-PCR on DCs stimulated over time (mean ± sd, N = 3); fold change was calculated by comparing the stimulated condition at various time points with the unstimulated control after normalization to the expression of the housekeeping gene.
(TIF)

Figure S6 **Clustering of Pathway Enrichment Factors (PEFs) obtained from the FET analysis on DC samples challenged with Curdlan for different times.** Pathway signatures were generated using DC-ATLAS pathways. Colored spots indicate significant (p≤0.05) up- (red) or down- (green) regulation. The colors of the dendrogram indicate the percentages of the tree support (significance), from 50% (pink) to 100% (black).
(TIF)

Figure S7 **Changes in expression of the genes in the Dectin-1 pathway upon 5-minutes Curdlan stimulation.** Red-colored genes are up-regulated, while green are down-regulated. White genes are not affected.
(PDF)

Figure S8 **Changes in expression of the genes in the Dectin-1 pathway upon 15-minutes Curdlan stimulation.** Red-colored genes are up-regulated, while green are down-regulated. White genes are not affected.
(PDF)

Figure S9 **Changes in expression of the genes in the Dectin-1 pathway upon 30-minutes Curdlan stimulation.** Red-colored genes are up-regulated, while green are down-regulated. White genes are not affected.
(PDF)

Figure S10 **Changes in expression of the genes in the Dectin-1 pathway upon 60-minutes Curdlan stimulation.** Red-colored genes are up-regulated, while green are down-regulated. White genes are not affected.
(PDF)

Figure S11 **Changes in expression of the genes in the Dectin-1 pathway upon 120-minutes Curdlan stimulation.** Red-colored genes are up-regulated, while green are down-regulated. White genes are not affected.
(PDF)

Figure S12 **Changes in expression of the genes in the Dectin-1 pathway upon 240-minutes Curdlan stimulation.** Red-colored genes are up-regulated, while green are down-regulated. White genes are not affected.
(PDF)

Figure S13 **Changes in expression of the genes in the Dectin-1 pathway upon 4 h-** ***S. cerevisiae*** **stimulation.** Red-colored genes are up-regulated, while green are down-regulated. White genes are not affected.
(PDF)

Table S1 **Normalized microarray data sets used for the analysis.** Each data set was normalized separately.
(7Z) Click here for additional data file.

Table S2 **Common and unique differentially expressed genes (DEGs) among the different cell wall stimuli versus the unstimulated control.**
(XLS)
