# Supplementary figures and images for: The Modular Nature of Dendritic Cell Responses to Commensal and Pathogenic Fungi
Source: PLoS One. 2012 Aug 3;7(8):e42430. doi: 10.1371/journal.pone.0042430 (PMC3411757; doi:10.1371/journal.pone.0042430)

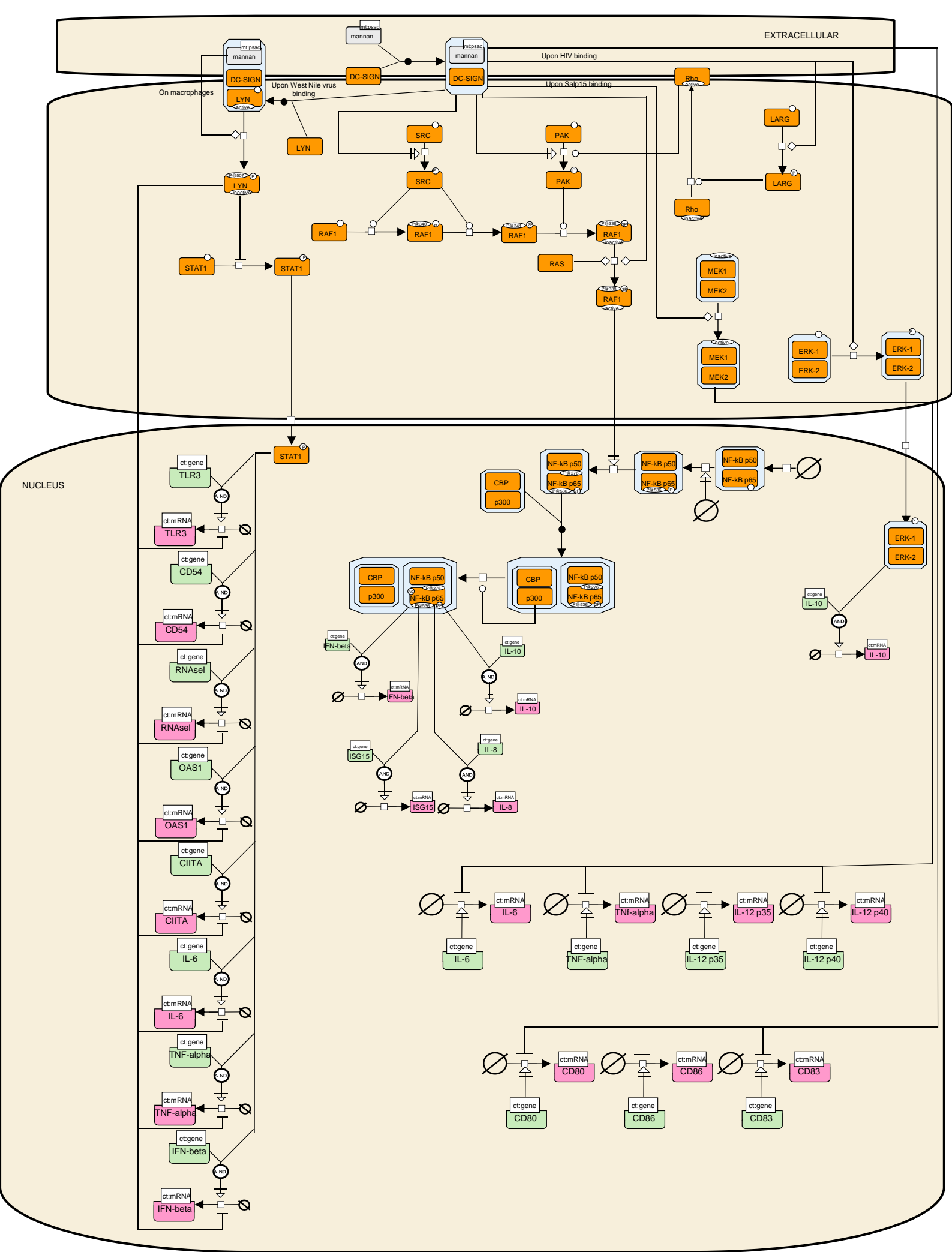

Supplement: Figure S1 — DC-SIGN map. The curation process lead to the reconstruction of signaling pathways. The map was drawn in an SBGN format. See Beltrame et al., 2011 and Cavalieri et al., 2010 for further details. (PDF) [file pone.0042430.s001.pdf]

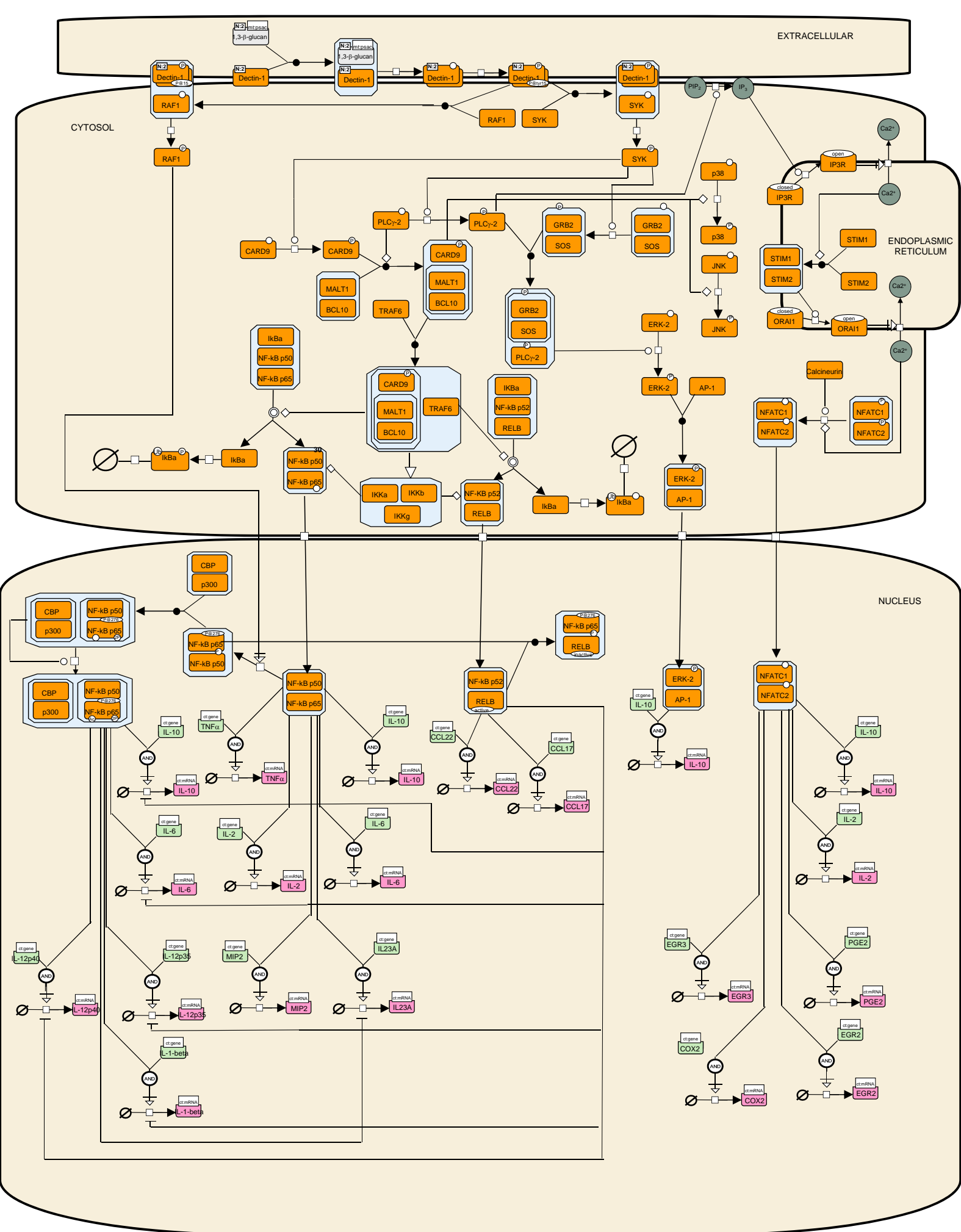

Supplement: Figure S2 — Dectin-1 map. The curation process lead to the reconstruction of signaling pathways. The map was drawn in an SBGN format. See Beltrame et al., 2011 and Cavalieri et al., 2010 for further details. (PDF) [file pone.0042430.s002.pdf]

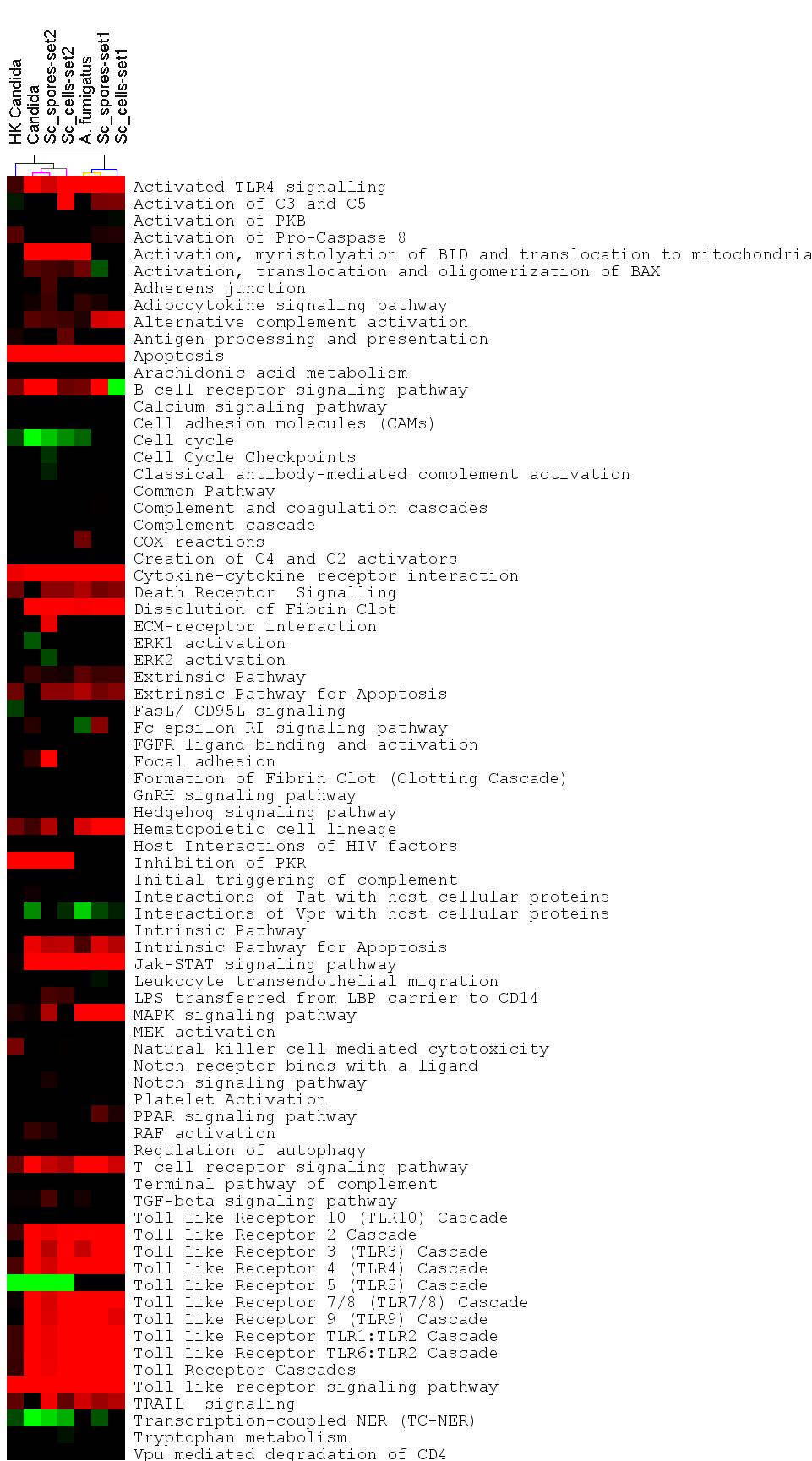

Supplement: Figure S3 — Clustering of Pathway Enrichment Factors (PEFs) obtained from the FET analysis on DC samples challenged with the different fungi. Pathway signatures were generated using immune-related public pathway available from KEGG and Reactome. Colored spots indicate significant (p≤0.05) up- (red) or down- (green) regulation. The colors of the dendrogram indicate the percentages of the tree support (significance), from 50% (pink) to 100% (black). (TIF) [file pone.0042430.s003.tif]

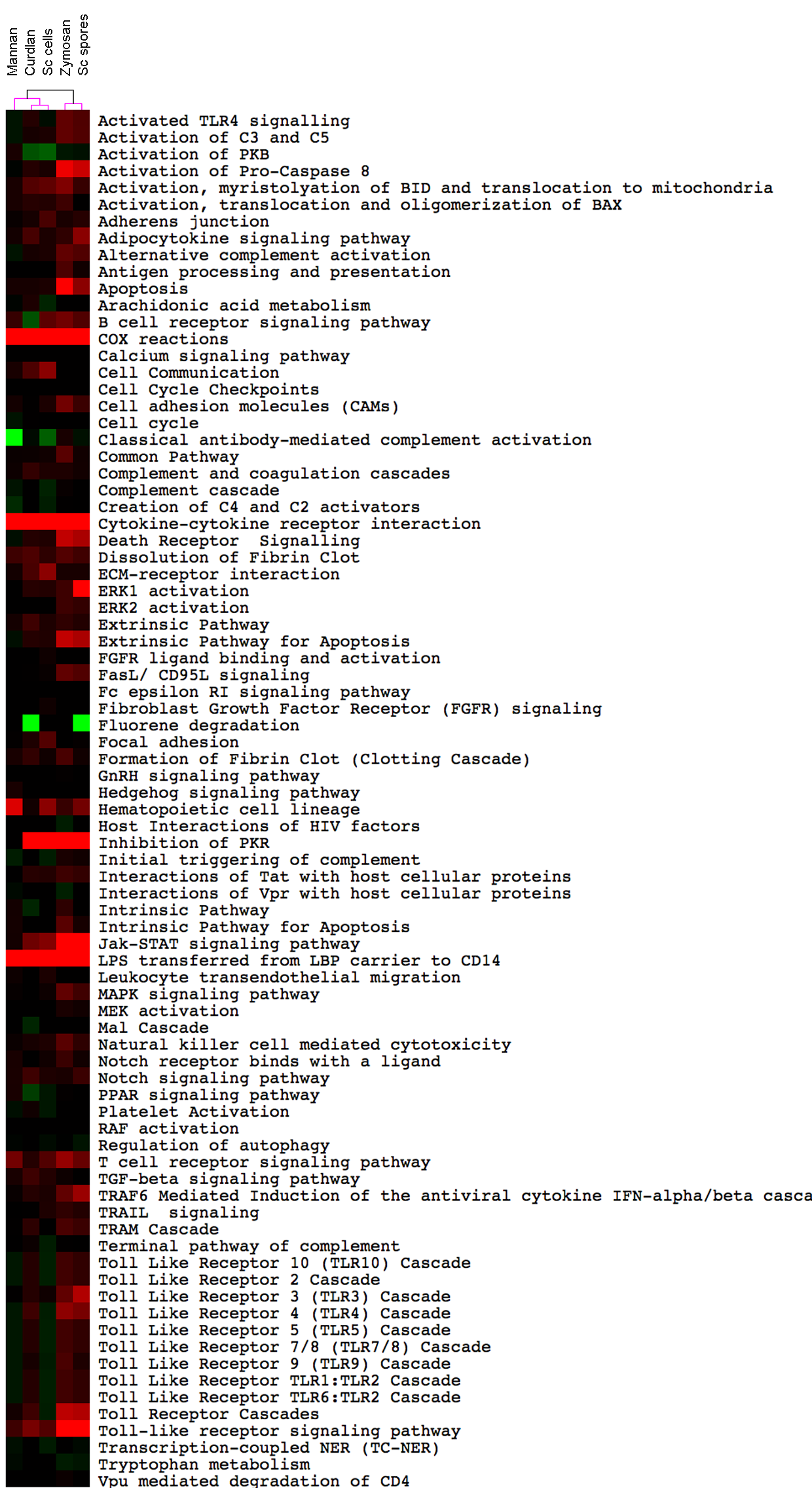

Supplement: Figure S4 — Clustering of Pathway Enrichment Factors (PEFs) obtained from the FET analysis on DC samples challenged with CLR-agonists. Pathway signatures were generated using immune-related public pathway available from KEGG and Reactome. Colored spots indicate significant (p≤0.05) up- (red) or down- (green) regulation. The colors of the dendrogram indicate the percentages of the tree support (significance), from 50% (pink) to 100% (black). (TIF) [file pone.0042430.s004.tif]

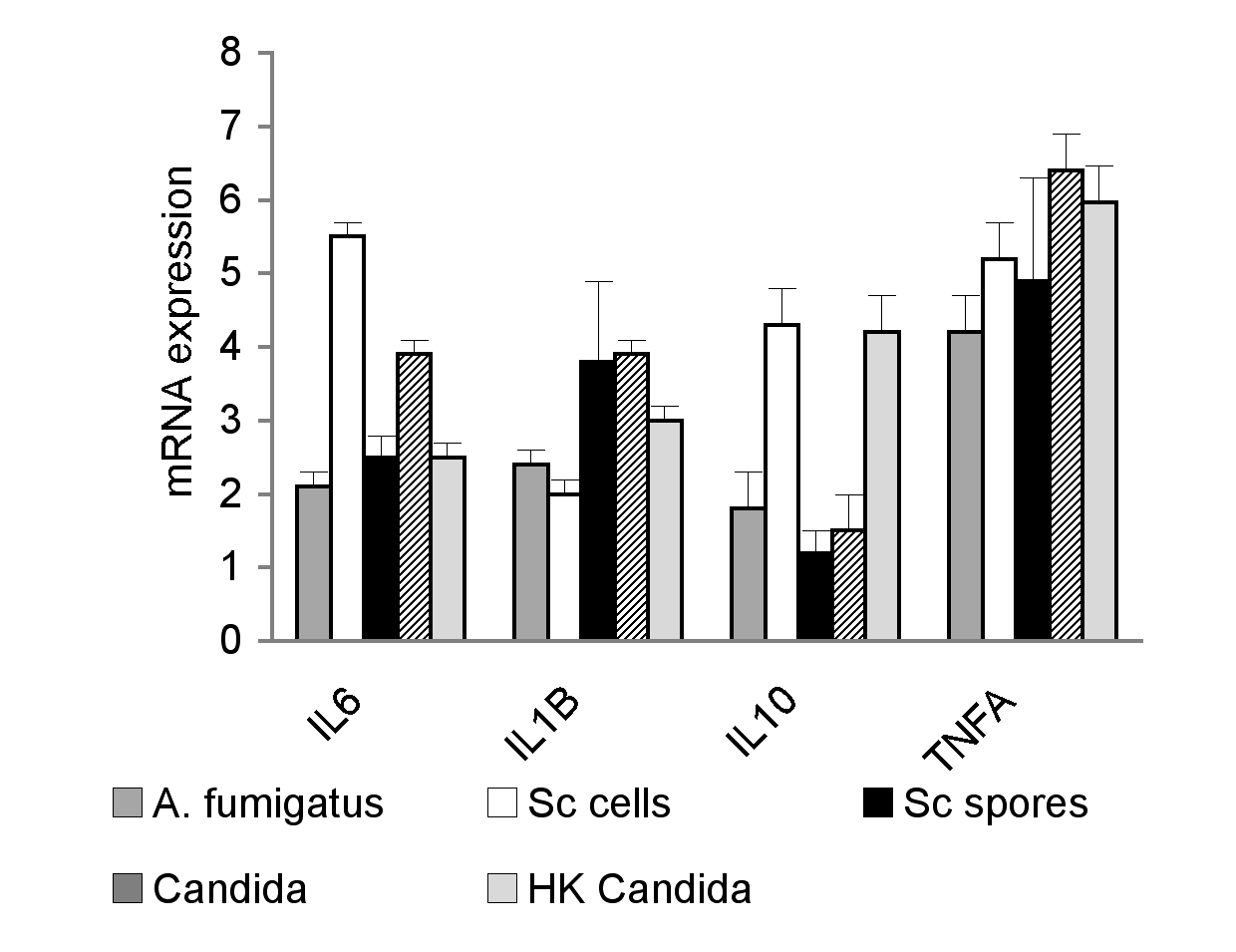

Supplement: Figure S5 — Changes in expression of cytokines at 4 h of stimulation. Cytokine gene expressions on DCs upon 4 hour-stimulation with A. fumigatus conidia, Sc cells and spores, heat-killed (HK) or live C. albicans hyphae. Gene expression was assessed in RT-PCR on DCs stimulated over time (mean ± sd, N = 3); fold change was calculated by comparing the stimulated condition at various time points with the unstimulated control after normalization to the expression of the housekeeping gene. (TIF) [file pone.0042430.s005.tif]

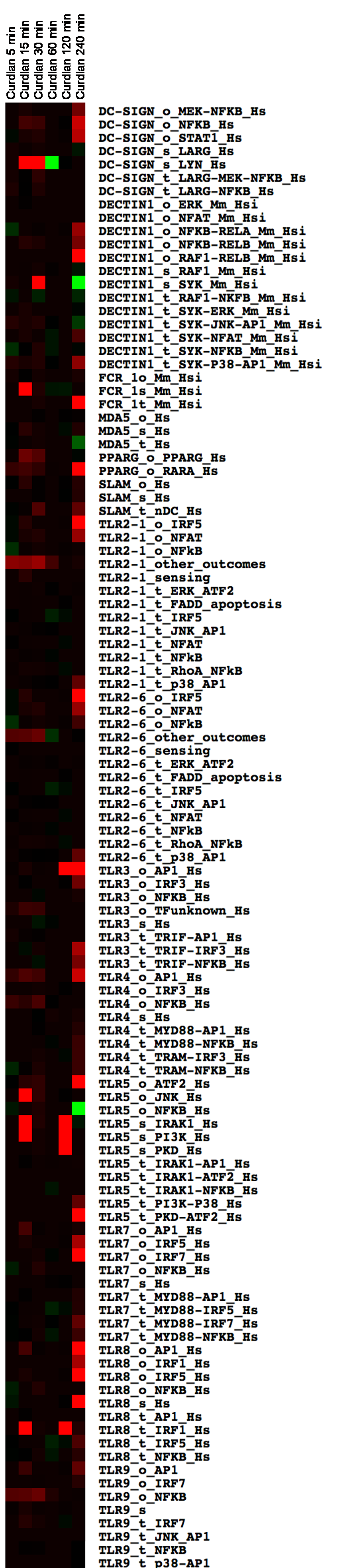

Supplement: Figure S6 — Clustering of Pathway Enrichment Factors (PEFs) obtained from the FET analysis on DC samples challenged with Curdlan for different times. Pathway signatures were generated using DC-ATLAS pathways. Colored spots indicate significant (p≤0.05) up- (red) or down- (green) regulation. The colors of the dendrogram indicate the percentages of the tree support (significance), from 50% (pink) to 100% (black). (TIF) [file pone.0042430.s006.tif]

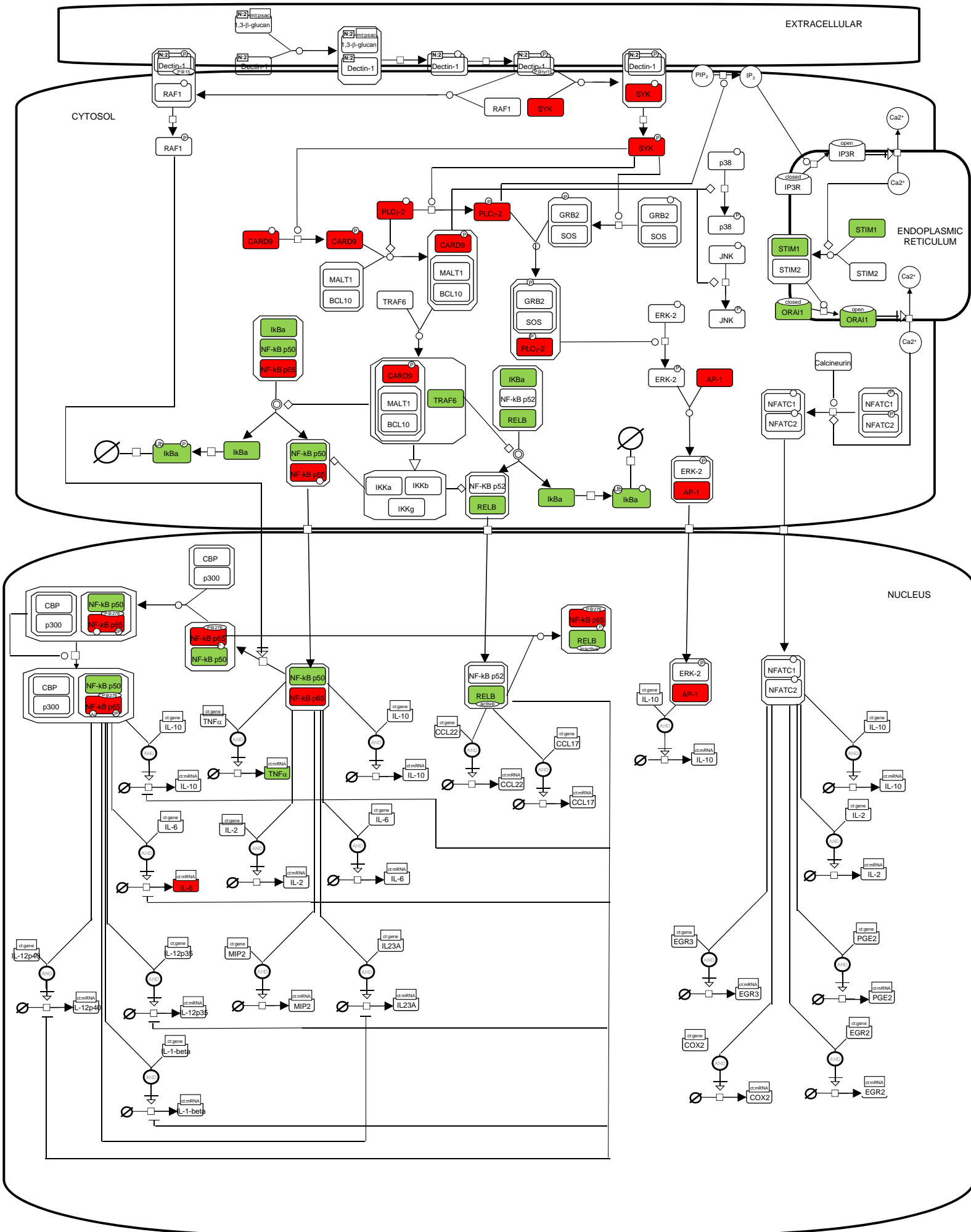

Supplement: Figure S7 — Changes in expression of the genes in the Dectin-1 pathway upon 5-minutes Curdlan stimulation. Red-colored genes are up-regulated, while green are down-regulated. White genes are not affected. (PDF) [file pone.0042430.s007.pdf]

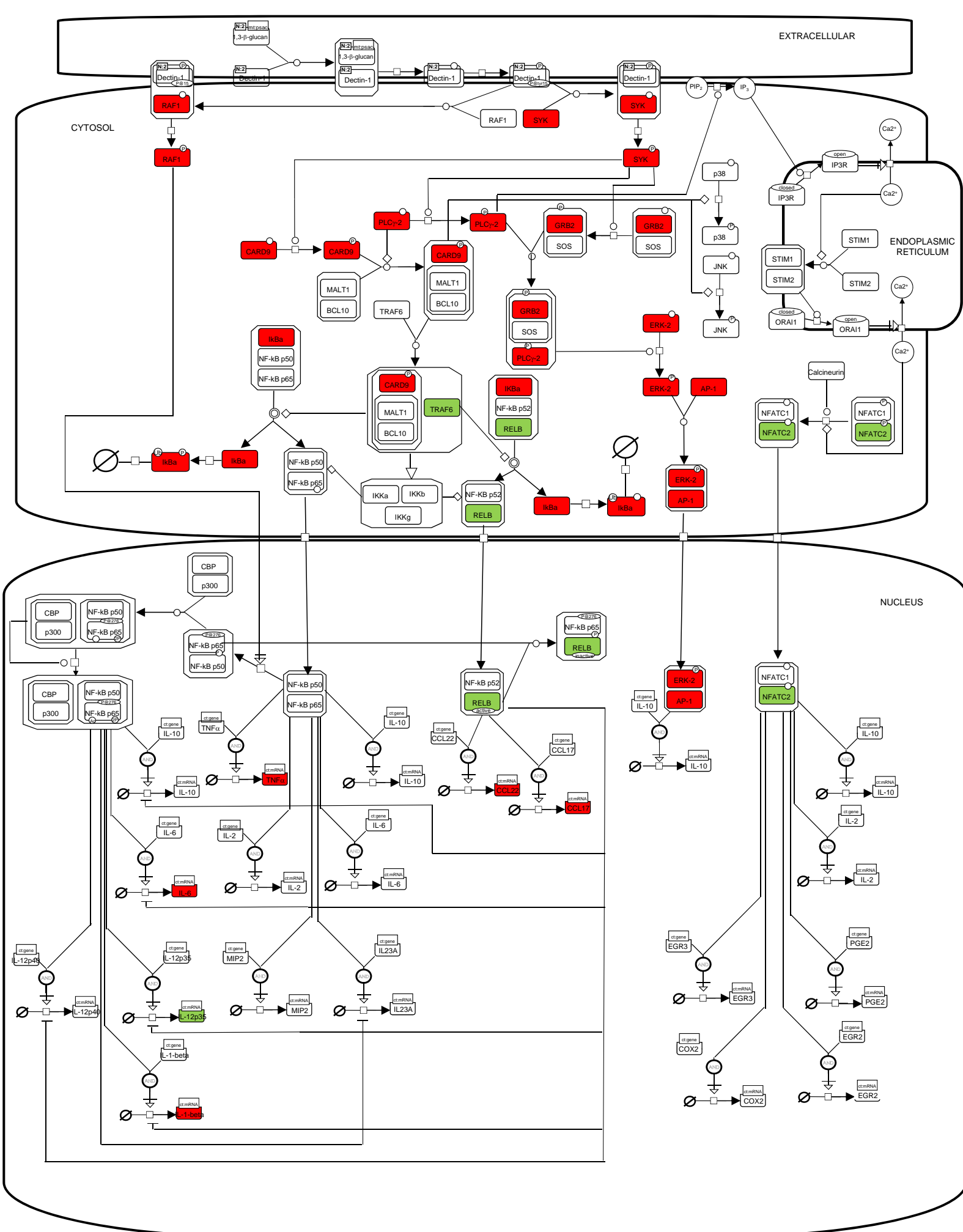

Supplement: Figure S8 — Changes in expression of the genes in the Dectin-1 pathway upon 15-minutes Curdlan stimulation. Red-colored genes are up-regulated, while green are down-regulated. White genes are not affected. (PDF) [file pone.0042430.s008.pdf]

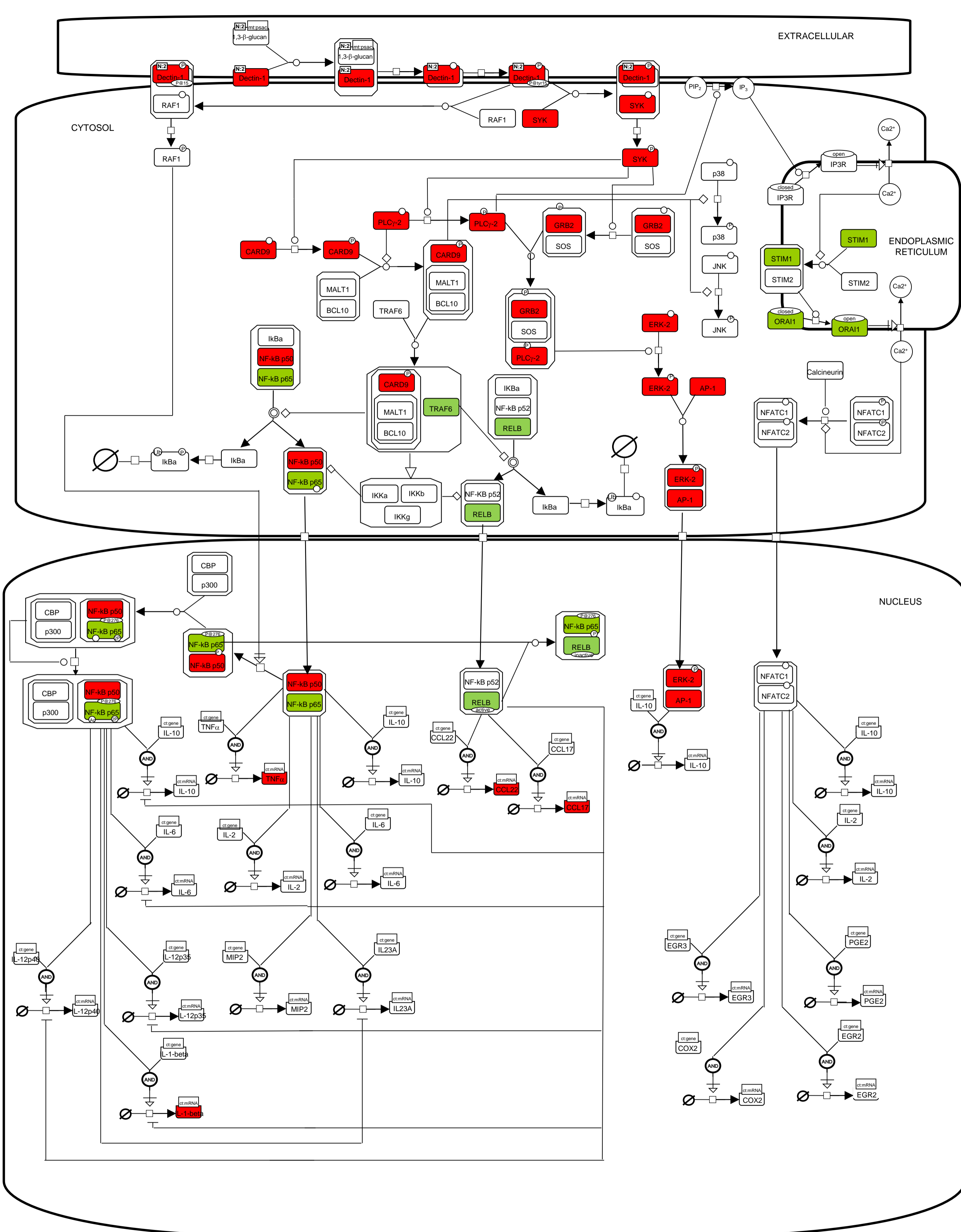

Supplement: Figure S9 — Changes in expression of the genes in the Dectin-1 pathway upon 30-minutes Curdlan stimulation. Red-colored genes are up-regulated, while green are down-regulated. White genes are not affected. (PDF) [file pone.0042430.s009.pdf]

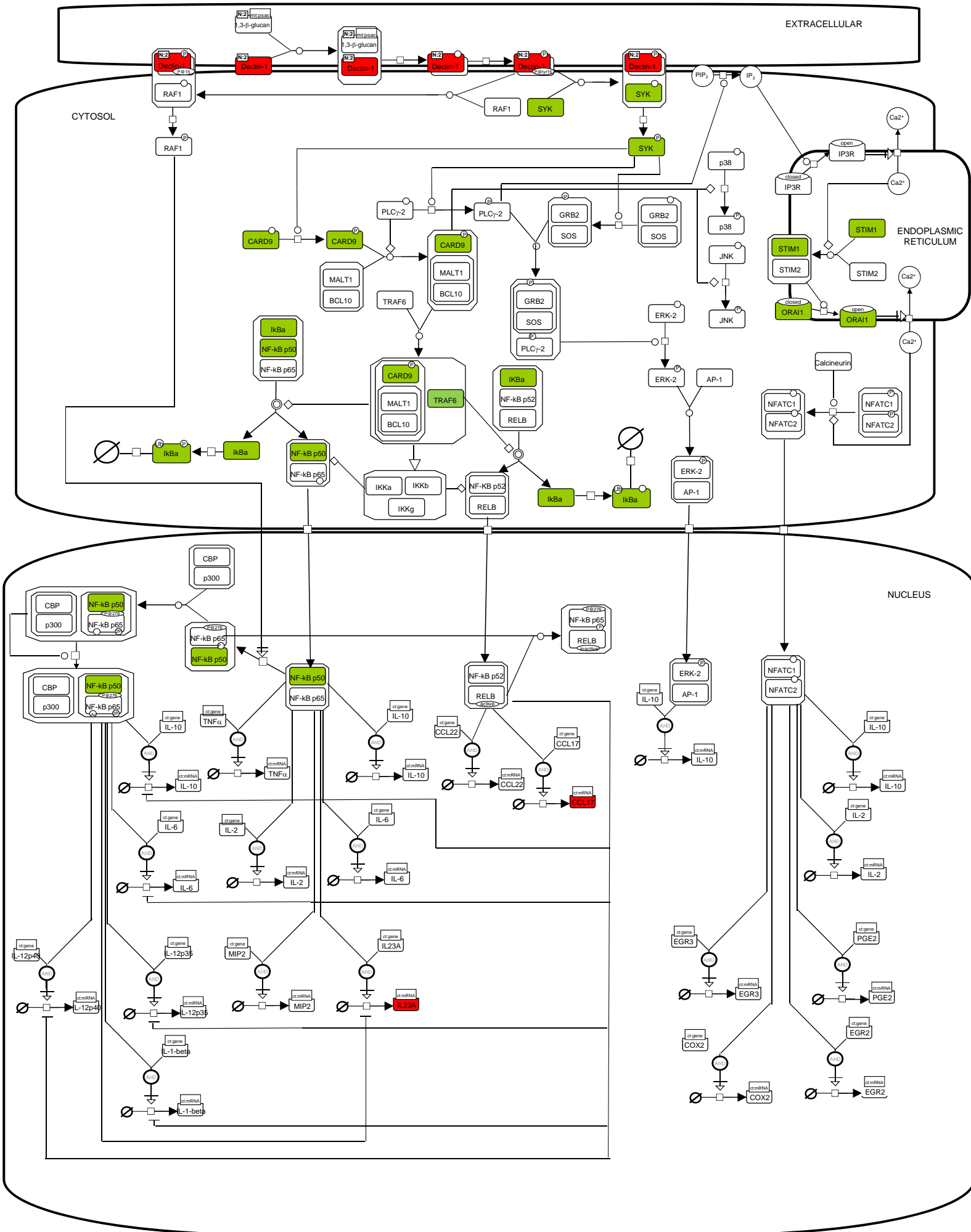

Supplement: Figure S10 — Changes in expression of the genes in the Dectin-1 pathway upon 60-minutes Curdlan stimulation. Red-colored genes are up-regulated, while green are down-regulated. White genes are not affected. (PDF) [file pone.0042430.s010.pdf]

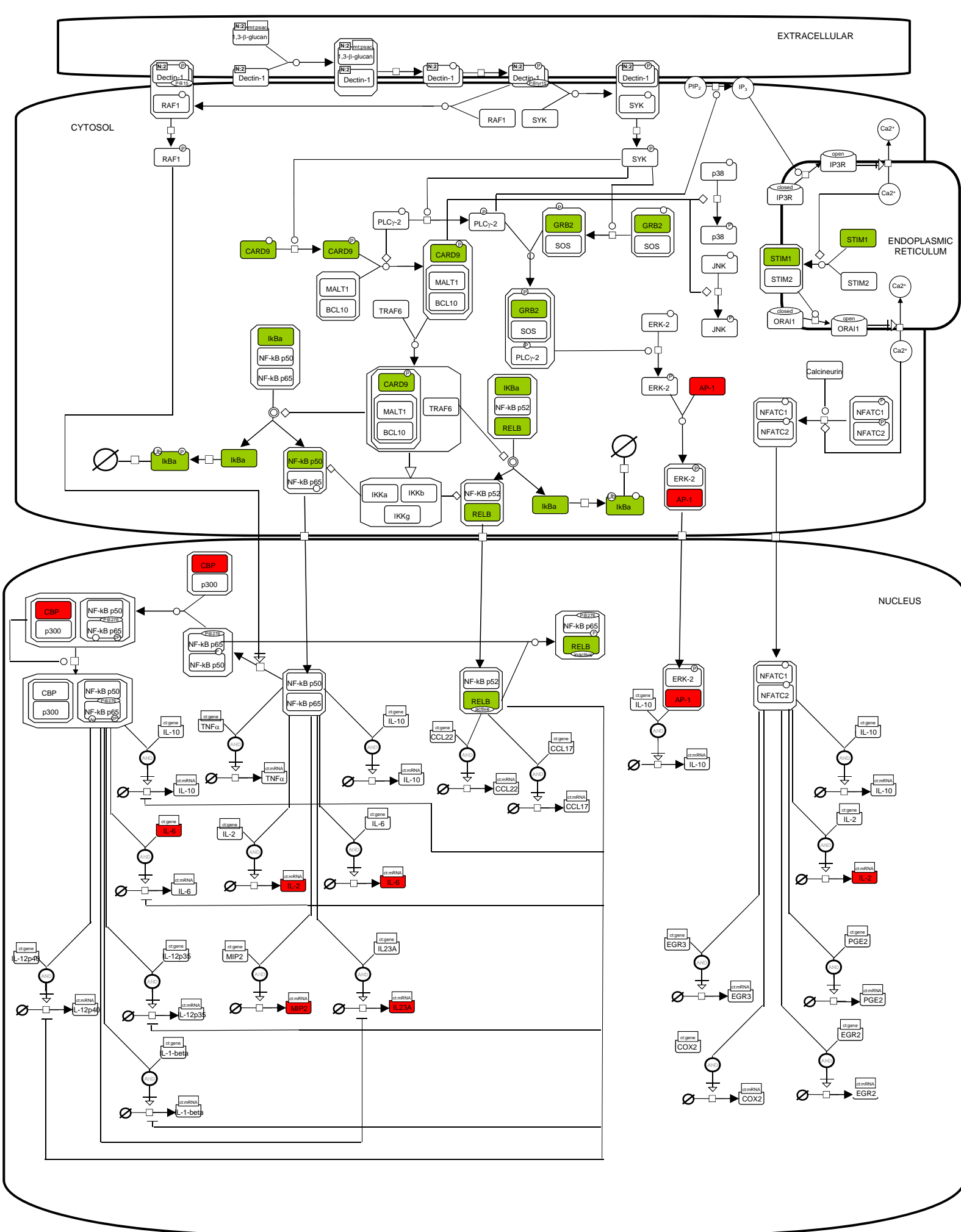

Supplement: Figure S11 — Changes in expression of the genes in the Dectin-1 pathway upon 120-minutes Curdlan stimulation. Red-colored genes are up-regulated, while green are down-regulated. White genes are not affected. (PDF) [file pone.0042430.s011.pdf]

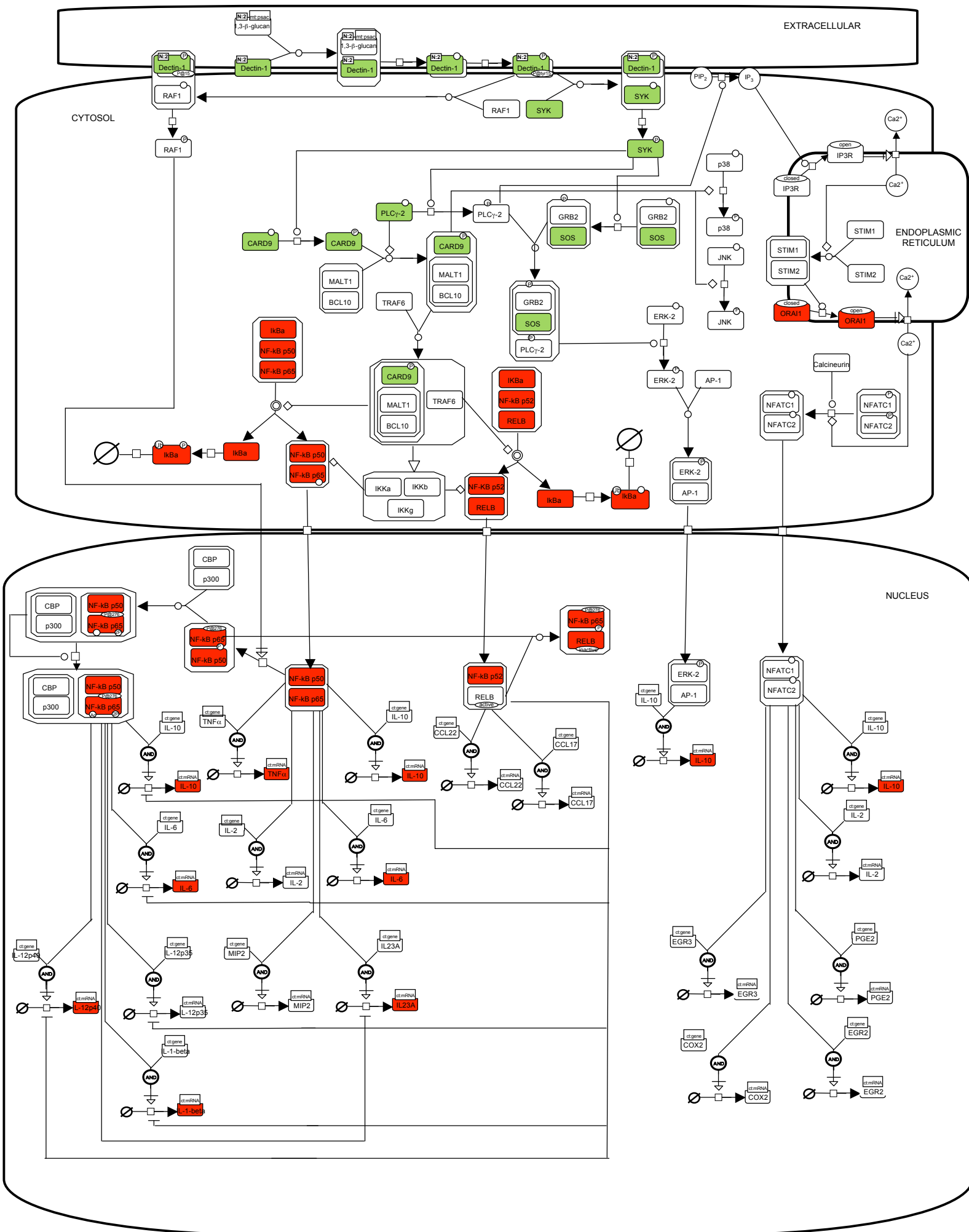

Supplement: Figure S12 — Changes in expression of the genes in the Dectin-1 pathway upon 240-minutes Curdlan stimulation. Red-colored genes are up-regulated, while green are down-regulated. White genes are not affected. (PDF) [file pone.0042430.s012.pdf]

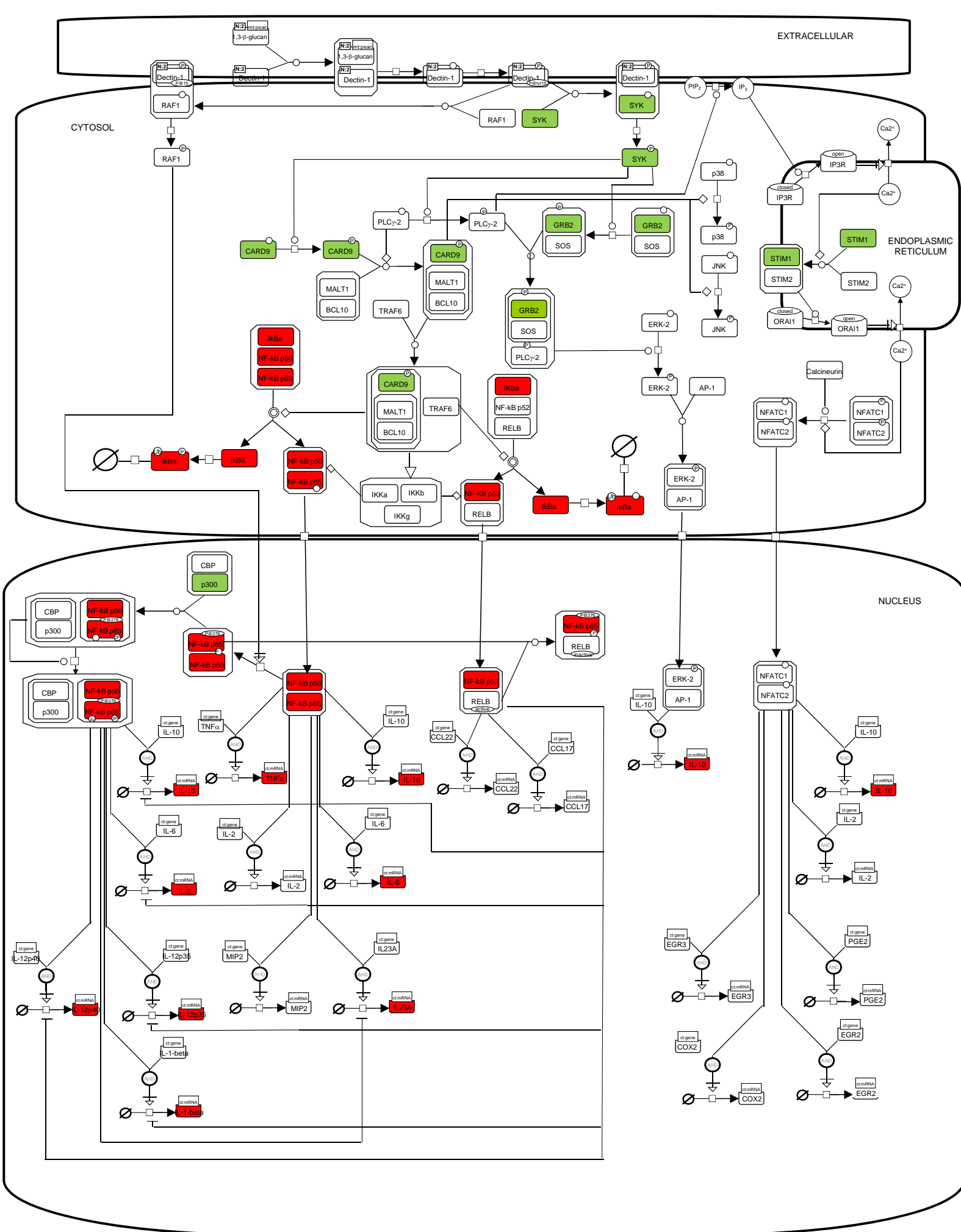

Supplement: Figure S13 — Changes in expression of the genes in the Dectin-1 pathway upon 4 h- S. cerevisiae stimulation. Red-colored genes are up-regulated, while green are down-regulated. White genes are not affected. (PDF) [file pone.0042430.s013.pdf]
